# Microorganism-regulated mechanisms of temperature effects on the performance of anaerobic digestion

**DOI:** 10.1186/s12934-016-0491-x

**Published:** 2016-06-03

**Authors:** Qiang Lin, Guihua He, Junpeng Rui, Xiaoyu Fang, Yong Tao, Jiabao Li, Xiangzhen Li

**Affiliations:** Key Laboratory of Environmental and Applied Microbiology, CAS; Environmental Microbiology Key Laboratory of Sichuan Province, Chengdu Institute of Biology, Chinese Academy of Sciences, Chengdu, 610041 China; University of Chinese Academy of Sciences, Beijing, 100049 People’s Republic of China

**Keywords:** Anaerobic digestion, Temperature gradient, Microbial diversity, Community structure, Potentially relative activity

## Abstract

**Background:**

Temperature is an important factor determining the performance and stability of the anaerobic digestion process. However, the microorganism-regulated mechanisms of temperature effects on the performance of anaerobic digestion systems remain further elusive. To address this issue, we investigated the changes in composition, diversity and activities of microbial communities under temperature gradient from 25 to 55 °C using 16S rRNA gene amplicon sequencing approach based on genomic DNA (refer to as “16S rDNA”) and total RNA (refer to as “16S rRNA”).

**Results:**

Microbial community structure and activities changed dramatically along the temperature gradient, which corresponded to the variations in digestion performance (e.g., daily CH_4_ production, total biogas production and volatile fatty acids concentration). The ratios of 16S rRNA to 16S rDNA of microbial taxa, as an indicator of the potentially relative activities in situ, and whole activities of microbial community assessed by the similarity between microbial community based on 16S rDNA and rRNA, varied strongly along the temperature gradient, reflecting different metabolic activities. The daily CH_4_ production increased with temperature from 25 to 50 °C and declined at 55 °C. Among all the examined microbial properties, the whole activities of microbial community and alpha-diversity indices of both microbial communities and potentially relative activities showed highest correlations to the performance.

**Conclusions:**

The whole activities of microbial community and alpha-diversity indices of both microbial communities and potentially relative activities were sensitive indicators for the performance of anaerobic digestion systems under temperature gradient, while beta-diversity could predict functional differences. Microorganism-regulated mechanisms of temperature effects on anaerobic digestion performance were likely realized through increasing alpha-diversity of both microbial communities and potentially relative activities to supply more functional pathways and activities for metabolic network, and increasing the whole activities of microbial community, especially methanogenesis, to improve the strength and efficiency in anaerobic digestion process.

**Electronic supplementary material:**

The online version of this article (doi:10.1186/s12934-016-0491-x) contains supplementary material, which is available to authorized users.

## Background

Anaerobic digestion (AD) is an effective process for converting organic waste, e.g., animal manure and food waste, into methane [1, 2]. Temperature and substrate are recognized to be the most important factors determining the performance and stability of the AD process [3–5]. Temperature affects AD performance mainly through shaping microbial community composition, activity and diversity, altering the biochemical conversion pathways and thermodynamic equilibrium of the biochemical reactions. The digestion process can be operated under mesophilic (35–40 °C) or thermophilic (55–60 °C) digesters. Thermophilic digestion increases degradation rates, and results in higher solid destruction and methane production [3]. The disadvantage of thermophilic digestion is the poor stability and reliability of the process [3, 6] and high cost of energy input. When other operation parameters are controlled, optimal performance of AD systems is likely compromised by the compound effects of temperature-dependent reaction rate and inhibition factors. Although the effects of temperature on AD performance have been investigated in many studies [7–9], the microorganism-regulated mechanisms behind such effects are still not systematically elucidated.

Microbial communities in AD systems are basically comprised of bacteria and archaea with a high complexity in terms of functionality and composition diversity [2, 4]. Each specific microorganism carries out one or more of four steps in AD food web, i.e., hydrolysis, acidogenesis, acetogenesis and methanogenesis [10]. Various microbial taxa respond to temperature at different rates and directions. Some methanogens had higher growth rates in thermophilic condition compared to mesophilic condition [1]. Although both hydrogenotrophic and acetoclastic methanogens exist in methanogenic systems, their preponderance in methanogenesis may shift with operation temperature [11]. For example, acetoclastic methanogenesis predominates at 35 °C, while hydrogenotrophic pathway is more important at 45 °C [12]. However, due to the complexity of microbial community, key microbial indicators for the performance of AD systems are not confidently proposed. There likely exists an optimal temperature, above which system performance would be seriously changed through the shifts in microbial diversity, community composition and activity. The relationships between temperature and microbial community composition, diversity and activity may differ at opposite side of this temperature threshold.

Estimating the metabolic activities of microbial communities in AD systems along temperature gradient is challenging. Most previous researches about the temperature effects on microbial community are based on 16S rDNA (refer to as 16S rRNA gene amplified from genomic DNA) which detect not only the living microorganisms but also the dormant and dead ones to represent the whole microbial community, resulting in lack of insight about the metabolic activity in situ [13–15]. Moreover, due to different ecological strategies for adaptation, microorganisms equip their genome with various number of rRNA genes [16], which result in biased enrichment of amplicon from the microbes with higher copies of 16S rDNA. Consequently, analysis only based on 16S rDNA is unable to present the actual state of a microbial community. Especially in functional systems, such as AD systems, microbial composition analysis is far from revealing system function which actually associates closely with active microbial populations. RNA molecules with extreme instability and much shorter lifetime compared with DNA, are used to indicate metabolically active microorganisms [14, 17, 18]. Thus, the analysis based on 16S rRNA (refer to as 16S rRNA gene amplified from total RNA) is capable to reveal potential activities of microbial community in situ. The relationship between activities of microbial community in situ and specific digestion performance (usually occurring at specific point in time) can be explored based on 16S rRNA datasets.

The ratios of 16S rRNA to 16S rDNA are presently used as in situ indicators for potentially relative activities (growth rates) in natural communities [14, 19]. In addition, the whole activities of microbial community are able to be mainly assessed by the similarity [18] between microbial communities based on 16S rDNA and rRNA datasets, and this is further supported by observed close correlations between microbial communities based on 16S rDNA and rRNA datasets [15, 19]. However, there are fewer studies to investigate the relationships between changes in microbial community composition, diversity, activity and performance under a temperature gradient by combining 16S rRNA and 16S rDNA approaches in AD systems, even though they may provide new and more actual insights to explore the relationships between shifts of microbial communities and system functions.

In this study, using 16S rRNA and 16S rDNA amplicon sequencing technique, we investigated the changes in microbial community properties under a temperature gradient from 25 to 55 °C, and their relationships with the performance of anaerobic digestion of swine manure which is widely used as substrate in AD systems. Particularly, we evaluated (i) the temperature-related shifts in microbial community composition and diversity, potentially relative activities of specific taxa in situ, whole activities of microbial community, and (ii) the microbial variables which are able to indicate the performance of AD systems under temperature gradient.

## Results and discussions

### Digestion performance

**Fig. 1:**
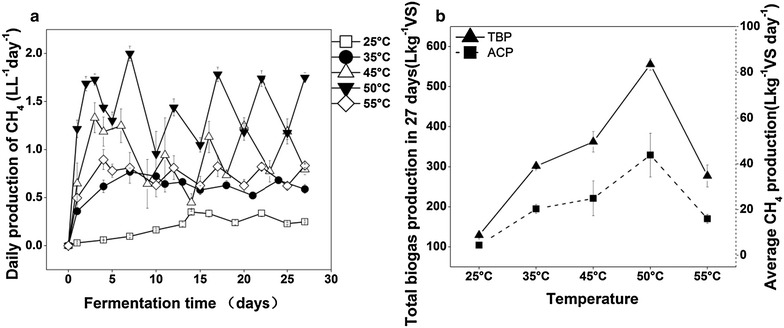
Digestion performance at different temperatures. The displayed volume of the gas has been normalized at standard temperature (273 K) and pressure (101325 Pa). **a** Daily CH_4_ production during 27 days of anaerobic fermentation at different temperature. **b** Total biogas production in 27 days (L kg^−1^ VS) and average CH_4_ production (L kg^−1^VS day^−1^) from initial period to peak period; *TBP* total biogas production in 27 days, *ACP* average CH_4_ production from initial period to peak period. All the data are presented as means ± standard deviations (n = 3)

After anaerobic digestion started, the days reaching the first peak of daily CH_4_ production (DCP) varied with temperature (Fig. 1a). For the first peak, the DCP was highest at 50 °C (1.7 L L^−1^ day^−1^), while it was lowest at 25 °C (0.35 L L^−1^ day^−1^). Average CH_4_ production (ACP) from initial period to first peak, determined by both methane content and biogas production, increased linearly with temperature from 25 to 50 °C, but decreased at 55 °C. The ACP reached up to 43.9 L kg^−1^ VS (volatile solid) day^−1^ at 50 °C, followed by those at 45, 55 and 35 °C, and it was only 4.5 L kg^−1^ VS at 25 °C (Fig. 1b). After the first peak, the DCP showed dynamic equilibrium until the end of fermentation. Total biogas production (TBP) within 27 days reached up to 555.6 L kg^−1^ VS at 50 °C, followed by 362.2 L kg^−1^ VS at 45 °C (Fig. 1b). Besides the DCP, the ACP and TBP also showed that the highest degradation efficiency of substrates for methane or biogas occurred at 50 °C. In addition, CH_4_ content in the biogas increased when temperature elevated from 25 to 45 °C, and was stable at higher temperatures, around 60 % in stable period (Additional file 1: Fig. S1). However, H_2_ content in biogas was low (0.1–0.8 %) during whole fermentation process. Because the DCP, TBP and ACP showed similar changing patterns with temperature, the DCP was selected as an indicator of AD performance.

The detected volatile fatty acids (VFAs) mainly contained acetic acid, propionic acid and butyric acid (Additional file 2: Table S3). Acetic acid concentration reduced with the digestion process, and maintained at 2–4 mM during stable period at all temperatures. Generally, standing acetic acid level from initial period to the first peak period was lowest at 50 °C, followed by that at 55 °C, and highest at 25 °C. The dynamic changes of butyric acid and propionic acid showed similar patterns with that of acetic acid when temperature was from 35 to 55 °C. However, at 25 °C, butyric acid and propionic acid accumulated up to 37 and 22 mM, respectively. The dynamics of VFAs generally showed that the conversion rates of VFAs were highest at 50 °C, and lowest at 25 °C. The VFAs concentrations showed negative correlations with DCP (Additional file 2: Table S4), indicating that the conversion rates of VFAs were important to methane production. Overall, at all temperatures except for 25 °C, there were no excessive accumulations of VFAs, thus, the microbial activities were unlikely inhibited.

The pH value was 7.0 at beginning and increased gradually until stable period (7.4–7.8) at different temperatures. The pH might be not important in shifting microbial community. The concentrations of NH_4_^+^-N increased throughout the process at each temperature (Additional file 2: Table S3). Overall, the concentrations of NH_4_^+^-N increased with temperature from 25 °C (18 mM) to 55 °C (43 mM). The increases of pH and the concentrations of NH_4_^+^-N throughout the process were probably due to the VFAs decrease and more free ammonia release with anaerobic digestion of swine manure.

### Overall changes of microbial community structure

**Fig. 2:**
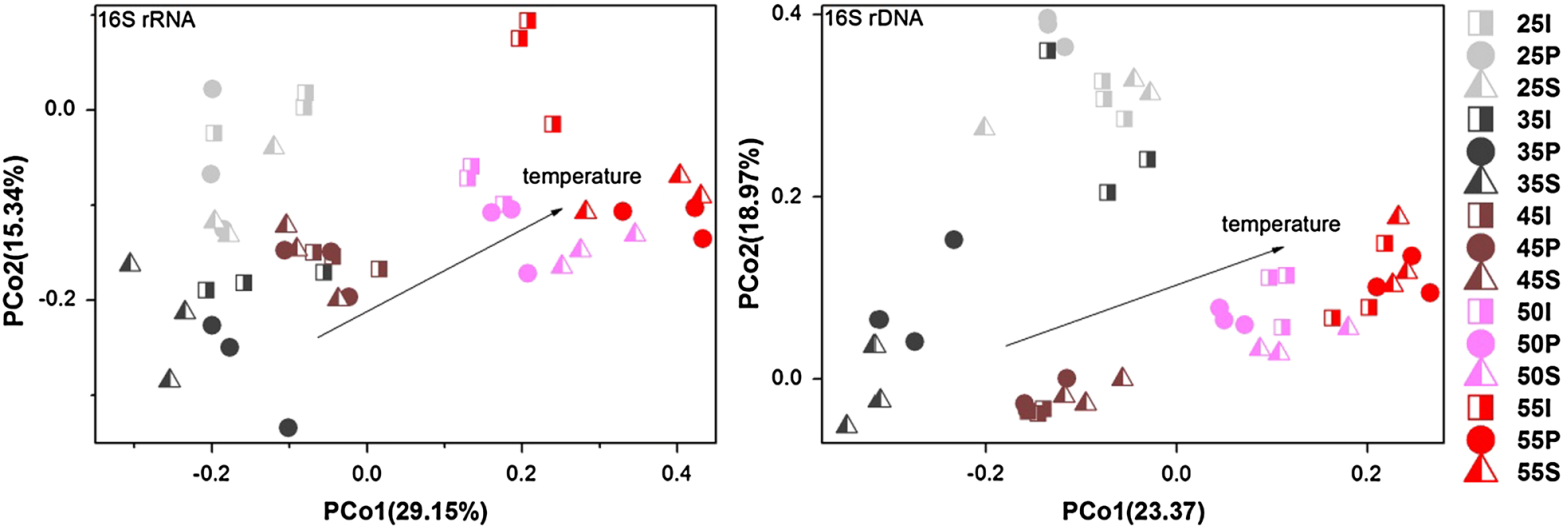
The principal coordinates analysis (PCoA) based on microbial community structure at different temperatures. “*I*”, “*P*”, “*S*” stand for initial, peak and stable period, respectively; the numbers mean different temperatures

Rarefaction curves for all the samples were close to plateau (Additional file 1: Fig. S2), indicating that the diversities of microbial communities were well captured. Principal coordinates analysis (PCoA) and PERMANOVA tests showed significant variations (*p* < 0.001) of microbial communities at different temperatures based on both 16S rDNA and 16S rRNA methods regardless of sampling periods (Fig. 2; Additional file 2: Table S5). Although high variations occurred within triplicate samples, the samples from each specific temperature usually formed a cluster, especially at high temperatures (45–55 °C), implicating that temperature was important to drive the shifts of microbial community. In addition, in consideration of the values of R^2^ and *p* in PERMANOVA tests in temperature and in digestion period, respectively, it further supported that compared to digestion period, temperature played more crucial role in shifting the abundance and activities of microbial community.

The variations of microbial communities expressed by Bray-Curtis distance based on both 16S rDNA and 16S rRNA datasets showed positive correlations with the variations of DCP and VFAs, respectively (n = 450, *p* < 0.01) (Additional file 1: Fig. S3), indicating that the variations of abundance and activities of microbial communities corresponded to the variations of system performances. Hence, the beta-diversity of a microbial community could predict the functional differences.

### Changes of microbial taxa

We assessed the abundance changes of microbial communities along temperature gradient based on 16S rDNA in peak and stable periods at different taxonomic ranks (Tables 1, 2). In the stable period, the phylum Firmicutes was dominant at all temperatures except for 35 °C. Among phylum Firmicutes, the genus *Clostridium* was abundant at 45 and 50 °C, and *Syntrophomonas* was abundant at 35 and 50 °C, and the relative abundances of genus *Pelotomaculum* were very low (0.01–0.07 %) at all temperature. The phylum Bacteroidetes was abundant at 25 to 45 °C. In the phylum Bacteroidetes, the genus *Ruminofilibacter* was more abundant at 35–50 °C, showing positive correlation with DCP. The genera *Prevotella* and *Bacteroides* were most abundant at 25 °C, showing negative correlations with DCP and temperature, respectively. The OTU84 (*Ruminofilibacter xylanolyticum*) as an only operational taxonomic unit (OTU) with average relative abundance >1 % under temperature gradient, was abundant at 45 and 50 °C and positively correlated with DCP (*p* < 0.05). The high abundance of OTU84 was probably related to the substrate and its wide growth temperature range. The relative abundances of Euryarchaeota were high at elevated temperature (from 45 to 55 °C). Among methanogens, genus *Methanosarcina* was abundant at 25, 45 and 50 °C. The relative abundances of *Methanobacterium* and *Methanoculleus* were high at elevated temperature from 50 to 55 °C. The relative abundances of Thermotogae (candidate genus S1 as main genus) were highest at 55 °C. The phyla WWE1 (candidate genus W22 as main genus) and Synergistetes (*Aminobacterium* as main genus) were abundant at 35 and 45 °C.

**Table 1:** Relative abundances of main microbial populations in stable period and their correlations to the performances

	25 °C	35 °C	45 °C	50 °C	55 °C	Temperature	DCP	Acetic acid	Propionic acid	Butyric acid	NH_4_^+^-N
***Firmicutes***	57.72 ± 9.67a	30.41 ± 1.51b	52.99 ± 1.17a	55.67 ± 0.57a	51.19 ± 2.74a	0.119	0.168	−0.007	0.168	0.354	0.145
Unclassified Clostridiales	12.54 ± 2.28b	7.08 ± 0.37c	18.9 ± 0.81a	16.68 ± 0.93ab	15.81 ± 2.56ab	0.524*	0.496	0.460	−0.198	−0.146	0.605*
Unclassified Clostridiaceae	10.72 ± 3.9a	2.87 ± 0.47b	2.99 ± 0.95b	1.92 ± 0.45b	2.78 ± 0.96b	−0.628*	−0.575*	−0.548*	0.688**	0.755**	−0.802**
Unclassified Lachnospiraceae	9.99 ± 1.61a	1.66 ± 0.07b	3.36 ± 0.38b	1.84 ± 0.12b	2.09 ± 0.12b	−0.722**	−0.608*	−0.549*	0.863**	0.941**	−0.753**
*Clostridium*	2.12 ± 0.58bc	1.47 ± 0.28c	4.73 ± 1.01a	3.5 ± 0.39ab	2.03 ± 0.42bc	0.284	0.549*	0.411	−0.097	−0.143	0.348
Unclassified Clostridia	0.74 ± 0.17c	0.61 ± 0.04c	2.12 ± 0.25b	4.77 ± 0.28a	5.1 ± 0.73a	0.873**	0.611*	0.516*	−0.716**	−0.493	0.900**
Candidate order MBA08	0.39 ± 0.01b	0.27 ± 0.02b	1.18 ± 0.13b	4.79 ± 0.44a	6.03 ± 0.79a	0.839**	0.488	0.460	−0.703**	−0.462	0.860**
Unclassified Ruminococcaceae	1.93 ± 0.28b	3.42 ± 0.27a	1.41 ± 0.25bc	0.94 ± 0.02c	0.83 ± 0.12c	−0.642**	−0.477	−0.434	0.271	0.116	−0.676**
*Syntrophomonas*	0.12 ± 0.03c	2.79 ± 1.07a	0.84 ± 0.24bc	2.41 ± 0.07ab	1.11 ± 0.21bc	0.223	0.378	−0.142	−0.669**	−0.579*	0.256
Candidate order SHA-98	0.25 ± 0.04c	0.24 ± 0.01c	1.36 ± 0.17b	3 ± 0.43a	2.39 ± 0.42a	0.836**	0.748**	0.482	−0.701**	−0.512	0.879**
*Lactobacillus*	1.65 ± 0.19ab	1.03 ± 0.17ab	1.98 ± 0.57a	1.57 ± 0.16ab	0.9 ± 0.14b	−0.162	0.206	−0.156	0.322	0.190	−0.234
*Sedimentibacter*	1.38 ± 0.45b	0.51 ± 0.06c	2.47 ± 0.12a	1.47 ± 0.17b	0.22 ± 0.04c	−0.110	0.350	0.061	0.298	0.161	−0.117
*Tepidimicrobium*	0.11 ± 0.01c	0.13 ± 0.02c	0.56 ± 0.06b	0.55 ± 0.03b	0.95 ± 0.16a	0.872**	0.417	0.730**	−0.619*	−0.522*	0.934**
*Pelotomaculum*	0.01 ± 0b	0.02 ± 0.01b	0.01 ± 0b	0.05 ± 0.01a	0.07 ± 0a	0.723**	0.283	0.405	−0.646**	−0.438	0.793**
***Bacteroidetes***	24.52 ± 7.78ab	29.16 ± 1.91a	23.01 ± 0.36ab	12.84 ± 2.18bc	4.13 ± 0.63c	−0.702**	−0.316	−0.453	0.506	0.300	−0.813**
*Ruminofilibacter*	0.59 ± 0.08c	6.17 ± 1.16b	11.42 ± 1a	8.12 ± 1.75b	0.5 ± 0.07c	0.215	0.667**	0.329	−0.218	−0.474	0.220
OTU84	0.44 ± 0.06c	4.95 ± 0.93b	9.42 ± 0.95a	6.6 ± 1.66ab	0.3 ± 0.07c	0.210	0.655**	0.322	−0.209	−0.461	0.217
Unclassified Porphyromonadaceae	2.68 ± 1.31b	3.19 ± 0.56ab	5 ± 0.44a	1.73 ± 0.35b	1.08 ± 0.2b	−0.280	−0.005	0.186	0.364	0.051	−0.338
*Prevotella*	10.13 ± 0.91a	0.93 ± 0.14b	0.2 ± 0.04b	0.25 ± 0.02b	0.22 ± 0.04b	−0.816**	−0.696**	−0.708**	0.877**	0.980**	−0.819**
Unclassified Bacteroidales	1.07 ± 0.55b	4.97 ± 0.41a	0.38 ± 0.02b	0.39 ± 0.04b	0.48 ± 0.02b	−0.431	−0.366	−0.280	0.064	−0.130	−0.448
*Bacteroides*	5.87 ± 4.1	0.21 ± 0.07	0.07 ± 0.02	0.09 ± 0.02	0.1 ± 0.02	−0.531*	−0.458	−0.425	0.606*	0.664**	−0.793**
***Euryarchaeota***	1.7 ± 0.66ab	0.5 ± 0.06b	3.08 ± 0.78a	2.64 ± 0.54a	2.26 ± 0.56ab	0.419	0.456	0.293	−0.150	−0.150	0.571*
*Methanosarcina*	1.31 ± 0.61ab	0.05 ± 0.02b	2.16 ± 0.85a	1.33 ± 0.36ab	0.5 ± 0.17ab	0.019	0.254	−0.040	0.180	0.115	0.028
*Methanobacterium*	0.03 ± 0.02c	0.02 ± 0.01c	0.02 ± 0c	0.49 ± 0.12b	0.85 ± 0.22a	0.714**	0.287	0.419	−0.594*	−0.366	0.791**
*Methanobrevibacter*	0.04 ± 0.01b	0.12 ± 0.04b	0.68 ± 0.13a	0.17 ± 0.05b	0.1 ± 0.02b	0.233	0.423	0.549*	−0.036	−0.283	0.262
*Methanoculleus*	0.1 ± 0.02b	0.05 ± 0.03b	0.07 ± 0.02b	0.32 ± 0.04a	0.39 ± 0.08a	0.706**	0.379	0.357	−0.566*	−0.323	0.754**
***Synergistetes***	0.42 ± 0.09c	1.22 ± 0.26a	0.92 ± 0.1ab	0.62 ± 0.06bc	0.42 ± 0.06c	−0.123	0.119	0.180	−0.030	−0.388	−0.154
*Aminobacterium*	0.15 ± 0.02b	0.77 ± 0.14a	0.2 ± 0b	0.19 ± 0.03b	0.14 ± 0.04b	−0.289	−0.177	−0.126	−0.023	−0.283	−0.314
***Thermotogae***	0.36 ± 0.04c	0.25 ± 0.02c	0.89 ± 0.15c	11.95 ± 2.11b	24.14 ± 0.58a	0.788**	0.262	0.493	−0.642**	−0.406	0.797**
S1	0.32 ± 0.05c	0.25 ± 0.02c	0.88 ± 0.15c	11.93 ± 2.11b	24.1 ± 0.58a	0.789**	0.262	0.494	−0.643**	−0.407	0.798**
***WWE1***	1.34 ± 0.7c	14.21 ± 1.97a	7.68 ± 1.3b	0.56 ± 0.16c	0.41 ± 0.11c	−0.295	−0.142	0.068	0.066	−0.285	−0.307
W22	1.34 ± 0.7c	14.21 ± 1.97a	7.68 ± 1.3b	0.56 ± 0.16c	0.41 ± 0.11c	−0.295	−0.142	0.068	0.066	−0.285	−0.307

The data (average relative abundance in genus level >0.1 %) were shown,except for *Pelotomaculum*

The data were based on 16S rDNA datasets

All data are presented as means ± standard deviations; values with different letters in a row mean significant difference at *p* < 0.05; bold italic fonts stand for phylum, while non-bold fonts stand for genus and 1 OTU

*DCP* daily CH_4_ production

** Significant at *p* < 0.01; * Significant at *p* < 0.05

**Table 2:** Relative abundances of main microbial populations in peak period and their correlations to the performances

	25 °C	35 °C	45 °C	50 °C	55 °C	Temperature	DCP	Acetic acid	Propionic acid	Butyric acid	NH_4_^+^-N
***Firmicutes***	44.14 ± 2.38cd	32.46 ± 0.95ghd	52.98 ± 1.16b	68.97 ± 1.86a	49.78 ± 2.53bc	0.592*	0.785**	−0.322	−0.464	−0.272	0.558*
Unclassified *Clostridiales*	11.15 ± 2.4b	10.46 ± 1.6b	11.79 ± 3.05b	14.04 ± 0.81a	15.19 ± 2.48a	0.853**	0.45	−0.427	−0.816**	−0.438	0.809**
Unclassified *Clostridiaceae*	9.16 ± 3.56a	1.38 ± 0.54c	3.7 ± 2.63b	1.66 ± 0.93c	2.26 ± 0.84ba	−0.720**	−0.633*	0.915**	0.811**	0.955**	−0.747**
Unclassified *Lachnospiraceae*	5.41 ± 3.8a	1.92 ± 0.04c	1.89 ± 0.1c	1.75 ± 0.35c	2.39 ± 0.96bc	−0.733**	−0.761**	0.954**	0.735**	0.973**	−0.771**
*Lactobacillus*	1.41 ± 0.17b	1.62 ± 0.41b	1.98 ± 0.57b	13.67 ± 0.67a	1.31 ± 0.28b	0.372	0.749**	−0.331	−0.416	−0.299	0.343
*Clostridium*	1.01 ± 0.14c	1.58 ± 0.11bc	4.73 ± 1.01a	3.1 ± 0.19b	1.91 ± 0.23bc	0.5	0.773**	−0.580*	−0.255	−0.532*	0.537*
*Sedimentibacter*	0.55 ± 0.04c	1.48 ± 0.2b	2.47 ± 0.12a	1.54 ± 0.19b	0.16 ± 0.02c	0.048	0.588*	−0.423	0.09	−0.38	0.106
*Syntrophomonas*	0.07 ± 0.01b	0.98 ± 0.18a	0.84 ± 0.24a	1.06 ± 0.12a	1.38 ± 0.33a	0.890**	0.551*	−0.879**	−0.934**	−0.924**	0.902**
*Tepidimicrobium*	0.11 ± 0.02c	0.13 ± 0.02c	1.33 ± 0.07c	5.09 ± 0.21a	0.27 ± 0.05b	0.443	0.843**	−0.403	−0.426	−0.362	0.421
*Pelotomaculum*	0.02 ± 0.02a	0.03 ± 0.02b	0.02 ± 0.02a	0.01 ± 0.01a	0.01 ± 0.0a	−0.05	−0.587	−0.04	−0.047	−0.111	−0.640*
***Bacteroidetes***	39.35 ± 3.67a	33.81 ± 2.81ab	23.00 ± 0.35bc	8.57 ± 0.49e	4.85 ± 0.12e	−0.967**	−0.666**	0.675**	0.921**	0.685**	−0.943**
*Ruminofilibacter*	0.73 ± 0.05c	5.8 ± 1.54bc	11.42 ± 1a	8.12 ± 1.75b	0.54 ± 0.09c	0.234	0.739**	−0.552*	−0.094	−0.509	0.285
OTU84	0.5 ± 0c	5.5 ± 1.5b	11.8 ± 0.9a	5.8 ± 0.6b	0.4 ± 0.1c	0.177	0.619*	−0.508	−0.008	−0.468	0.236
Unclassified *Bacteroidales*	2.1 ± 1.67b	0.58 ± 0.06c	2.22 ± 1.69b	5.8 ± 3.79a	0.76 ± 0.34c	0.229	0.698**	−0.137	−0.188	−0.081	0.198
*Prevotella*	23.98 ± 3.9a	1.58 ± 0.34b	0.2 ± 0.04b	0.38 ± 0.05b	0.19 ± 0.02b	−0.818**	−0.721**	0.965**	0.806**	0.989**	−0.852**
*Bacteroides*	5.05 ± 0.59a	0.4 ± 0.09b	0.07 ± 0.02b	0.21 ± 0.05b	0.12 ± 0.01b	−0.814**	−0.716**	0.964**	0.800**	0.988**	−0.849**
Unclassified *Porphyromonadaceae*	2.46 ± 0.5b	7.76 ± 3.17a	0.68 ± 0.22b	0.57 ± 0.01b	1.66 ± 0.32b	−0.475	−0.487	0.064	0.233	0.006	−0.441
***Euryarchaeota***	1.09 ± 0.16b	1.01 ± 0.23b	3.23 ± 0.78ab	4.06 ± 0.68a	3.50 ± 0.96ab	0.903**	0.825**	−0.639*	−0.760**	−0.617*	0.886**
*Methanosarcina*	0.73 ± 0.17ab	0.07 ± 0.03b	2.16 ± 0.85a	1.84 ± 0.43ab	1.47 ± 0.69ab	0.670**	0.727**	−0.416*	−0.41	−0.364	0.664**
*Methanoculleus*	0.06 ± 0.02b	0.03 ± 0.01b	0.07 ± 0.02b	0.1 ± 0.02b	0.27 ± 0.03a	0.719**	0.072	−0.281	−0.688**	−0.315	0.683**
*Methanobrevibacter*	0.13 ± 0.02b	0.23 ± 0.08b	0.68 ± 0.13ab	1.08 ± 0.17a	0.22 ± 0.08b	0.5	0.936**	−0.529*	−0.409	−0.48	0.499
*Methanobacterium*	0.01 ± 0c	0.02 ± 0.01c	0.02 ± 0c	0.63 ± 0.19b	1.02 ± 0.48a	0.808**	0.32	−0.42	−0.844**	−0.451	0.764**
***Thermotogae***	0.26 ± 0.01b	0.26 ± 0.07b	0.88 ± 0.15b	1.78 ± 0.29b	24.64 ± 4.55a	0.643**	−0.066	−0.266	−0.644**	−0.314	0.616*
S1	0.24 ± 0c	0.24 ± 0.08c	0.88 ± 0.15c	1.76 ± 0.29c	24.61 ± 4.56a	0.818**	0.223	−0.421	−.802**	−0.455	0.616*
***WWE1***	0.47 ± 0.05b	4.82 ± 1.46ab	7.67 ± 1.30a	0.61 ± 0.18b	0.32 ± 0.12b	−0.06	0.203	−0.342	0.2	−0.327	0.015
W22	0.47 ± 0.05b	4.82 ± 1.47ab	7.67 ± 1.3a	0.61 ± 0.18b	0.32 ± 0.01b	−0.06	0.203	−0.342	0.2	−0.327	0.015
***Synergistetes***	0.61 ± 0.07b	2.45 ± 0.83a	0.92 ± 0.09b	0.55 ± 0.07b	0.39 ± 0.06b	−0.377	−0.2	−0.156	0.203	−0.196	−.322
*Aminobacterium*	0.4 ± 0.06b	1.54 ± 0.64a	0.2 ± 0b	0.11 ± 0.03b	0.1 ± 0.01b	−0.504	−0.391	0.027	0.284	−0.02	−0.462

The data (average relative abundance in genus level >0.1 %) were shown,except for *Pelotomaculum*

The data were based on 16S rDNA datasets

All data are presented as means ± standard deviations; values with different letters in a row mean significant difference at p < 0.05; bold italic fonts stand for phylum, while non-bold fonts stand for genus and 1 OTU

*DCP* daily CH4 production

** Significant at *p* < 0.01; * Significant at *p* < 0.05

In the peak period, overall, the changing trends of these core taxa were similar with those in the stable period. However, the difference occurred in phylum Firmicutes which was most abundant at 50 °C, followed by that at 45 °C. The relative abundance of Firmicutes showed positive correlation with both temperature and DCP (*p* < 0.01). *Lactobacillus* was most abundant at 50 °C, followed by 45 °C. The relative abundances of *Methanosarcina* were higher when temperature was above 45 °C, and positively correlated with temperature, DCP and NH_4_^+^-N. This was probably attributed to the wide adaptation of *Methanosarcina* to high temperature and NH_4_^+^-N concentrations [20]. In addition, *Methanosarcina* negatively correlated with acetic acid, mainly due to its possessing acetoclastic methanogenesis pathway [20].

In both peak and stable periods, the relative abundance of Firmicutes showed negative correlation with that of Bacteroidetes along temperature gradient (*p* < 0.01), indicating that many microbes in phylum Firmicutes may have competition relationship with those in phylum Bacteroidetes. This potential competition relationship between them probably resulted from the partial overlap of their ecological niches in AD systems [9]. In addition, the relative abundance of the phylum Firmicutes positively correlated with that of Euryarchaeota (*p* < 0.01), indicating the possible cooperation between them.

### Changes of potential microbial activities in situ

**Fig. 3:**
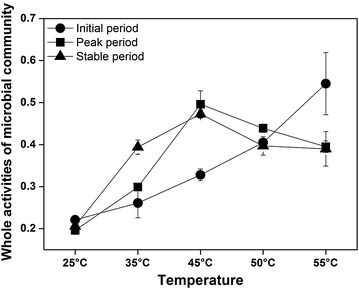
The whole activities of microbial communities at different temperatures. The whole activities in both peak and stable periods positively correlated with temperature and DCP (*p* < 0.01). All the data are presented as means ± standard deviations (n = 3)

The whole activities of microbial community were mainly assessed by the similarities [18] (based on Bray-Curtis distance) between microbial communities based on 16S rDNA and 16S rRNA datasets, respectively. The whole activities of microbial community were maximum at 45 °C, followed by that at 50 °C in both peak and stable periods, and they positively correlated with temperature and DCP (*p* < 0.01) (Fig. 3). The regression analysis between similarities and DCP showed a linear correlation (*p* < 0.01) (Additional file 1: Fig. S4), which indicated that whole activities of microbial community contributed much to DCP. In addition, based on the occurrence frequency of each OTU, in both stable (Additional file 1: Fig. S5) and peak periods (Additional file 1: Fig. S6) along the temperature gradient, the activities (16S rRNA frequencies) roughly corresponded to their abundances (16S rDNA frequencies). This agreed well with the previous report [19]. The relationships between activities and abundances were stronger at elevated temperature from 45 to 50 °C due to higher values of slope and R^2^. This further supported that higher whole activities of microbial community occurred at elevated temperatures especially at 45 and 50 °C.

The whole activities of functional and dominant phyla, such as Firmicutes, Bacteroidetes and Euryarchaeota, were further analyzed in detail. Overall, the whole activities of Firmicutes, Bacteroidetes and Euryarchaeota showed significant and positive correlation with DCP, respectively (Additional file 2: Table S7), and increased with temperature from 25 to 50 °C and decreased at 55 °C. The whole activities decreased at 55 °C, probably because high concentrations of NH_4_^+^-N or other factors inhibited microbial activities [21, 22]. Similar increase of the whole activities of main phyla indicated that all the core functional pathways were strengthened. Hence, the AD system might be prevented from collapse by eliminating the accumulation of inhibitory intermediates induced by inharmoniousness in metabolic activities and pathways. Consequently, one of mechanisms of temperature effects on AD performance was probably realized through regulating the whole activities of microbial community.

Potentially relative activity was assessed by the ratio of 16S rRNA to rDNA for a specific taxon [15, 17, 19], which was calculated by (rRNA/[rDNA + 1]) [23]. In the stable period (Table 3), the OTU84 was active at 25 °C. The potentially relative activity of genus *Clostridium* showed positive correlation with temperature, but it was lower <1.0 at all temperature (25–55 °C). The genus *Lactobacillus* was more active at 55 °C, and the potentially relative activities at all temperatures (25–55 °C) were above 1.0. The potentially relative activity of genus *Syntrophomonas* was low, and it showed positive correlations with VFAs. The genus *Pelotomaculum* was most active at 50 °C with potentially relative activity of 4.2, which positively correlated with DCP. The genus *Ruminofilibacter* was more active at 55 °C. The genus S1 was active at 25 and 35 °C. *Aminobacterium* was active at all temperatures except for 45 °C. Among methanogens, *Methanosarcina* was most active at all temperatures (25–55 °C), and more active at 50 and 55 °C. The two other methanogens *Methanobrevibacter* and *Methanobacterium* had low potentially relative activities. *Methanoculleus* was very active under all temperatures.

**Table 3:** The relative activities of specific populations in stable period and their correlations to the performances

	25 °C	35 °C	45 °C	50 °C	55 °C	Temperature	DCP	Acetic acid	Propionic acid	Butyric acid	NH_4_^+^-N
***Firmicutes***											
Unclassified Clostridiales	0.63 ± 0.12	0.61 ± 0.1	0.47 ± 0.02	0.5 ± 0.03	0.44 ± 0.02	−0.569*	−0.371	−0.390	0.523*	0.379	−0.943**
Unclassified Clostridiaceae	0.09 ± 0.04b	0.18 ± 0.05b	0.73 ± 0.29ab	1.41 ± 0.43a	1.24 ± 0.39a	0.736**	0.615*	0.472	−0.601*	−0.457	0.861**
Unclassified Lachnospiraceae	0.24 ± 0.05b	0.99 ± 0.11a	0.32 ± 0.03b	0.43 ± 0.04b	0.44 ± 0.06b	−0.058	−0.068	−0.003	−0.274	−0.468	−0.070
*Clostridium*	0.22 ± 0.05c	0.51 ± 0.14ab	0.44 ± 0.06bc	0.54 ± 0.08ab	0.75 ± 0.06a	0.712**	0.332	0.599*	−0.630*	−0.676**	0.878**
Unclassified Clostridia	1.08 ± 0.23a	0.82 ± 0.07ab	1.13 ± 0.09a	0.6 ± 0.06b	0.67 ± 0.06b	−0.489	−0.364	−0.085	0.662**	0.433	**−**0.614*
Candidate order MBA08	0.76 ± 0.06c	1.44 ± 0.08a	1 ± 0.07b	0.78 ± 0.06c	0.93 ± 0.06bc	−0.120	−0.182	0.007	−0.225	−0.433	**−**0.125
Unclassified Ruminococcaceae	1.24 ± 0.27a	0.39 ± 0.07b	0.54 ± 0.07b	0.64 ± 0.06b	0.61 ± 0.11b	−0.477	−0.406	−0.497	0.640*	0.772**	**−**0.578*
*Syntrophomonas*	0.91 ± 0.3a	0.06 ± 0.03b	0.22 ± 0.07b	0.08 ± 0.02b	0.22 ± 0.07b	−0.581*	−0.569*	−0.436	0.727**	0.825**	**−**0.305
Candidate order SHA-98	1.05 ± 0.07	1.53 ± 0.56	0.81 ± 0.11	0.68 ± 0.14	1.51 ± 0.25	−0.038	−0.398	−0.164	−0.205	−0.087	−0.047
*Lactobacillus*	1.3 ± 0.15b	2.33 ± 0.78b	7.91 ± 4.17ab	10.15 ± 2.01ab	16.51 ± 6.57a	0.693**	0.335	0.643**	−0.512	−0.398	0.945**
*Sedimentibacter*	0.18 ± 0.05b	0.31 ± 0.1b	0.15 ± 0.01b	0.15 ± 0.04b	0.61 ± 0.03a	0.415	−0.263	0.444	−0.303	−0.285	0.471
*Tepidimicrobium*	1.24 ± 0.32	0.76 ± 0.1	0.86 ± 0.1	1.15 ± 0.16	1.29 ± 0.29	0.110	−0.051	−0.099	0.044	0.210	0.191
*Pelotomaculum*	0.34 ± 0.34b	0.33 ± 0.33b	1.02 ± 0.01b	4.16 ± 1.08a	1.08 ± 0.9b	0.461	0.742**	0.106	−0.493	−0.320	0.533*
***Bacteroidetes***											
*Ruminofilibacter*	1.19 ± 0.16	1.03 ± 0.19	1.45 ± 0.25	0.86 ± 0.33	5.3 ± 4.87	0.278	−0.098	0.308	−0.171	−0.119	0.596*
OTU84	7.62 ± 6.74	1.15 ± 0.37	1.1 ± 0.36	1.01 ± 0.35	0.43 ± 0.22	−0.443	−0.348	−0.384	0.449	0.525*	−0.828**
Unclassified Porphyromonadaceae	0.27 ± 0.11	0.51 ± 0.05	0.23 ± 0.05	0.31 ± 0.13	0.36 ± 0.08	−0.039	−0.139	−0.168	−0.244	−0.248	−0.065
*Prevotella*	0.59 ± 0.16	1.43 ± 0.45	1.42 ± 0.25	1 ± 0.19	0.99 ± 0.15	0.197	0.216	0.443	−0.289	−0.485	0.300
Unclassified Bacteroidales	6.59 ± 2.6a	2.57 ± 0.55ab	2.89 ± 0.73ab	0.81 ± 0.09b	0.45 ± 0.07b	−0.725**	−0.539*	−0.603*	0.708**	0.698**	−0.920**
*Bacteroides*	0.76 ± 0.29b	11.2 ± 4.37a	2.44 ± 0.82b	1.21 ± 0.21b	0.83 ± 0.2b	−0.241	−0.156	−0.256	−0.216	−0.268	−0.299
***Euryarchaeota***											
*Methanosarcina*	2.66 ± 2.19b	6.05 ± 3.77b	2.69 ± 1.83b	9.4 ± 2.41b	18.54 ± 2.72a	0.628*	0.130	0.509	−0.551*	−0.391	0.747**
*Methanobacterium*	1.49 ± 0.79ab	0.11 ± 0.11b	2.9 ± 0.73a	1.44 ± 0.34ab	1.72 ± 0.42ab	0.262	0.254	0.420	0.081	0.043	0.355
*Methanobrevibacter*	1.79 ± 1.15	0.65 ± 0.34	0.34 ± 0.11	1.83 ± 0.4	1.15 ± 0.4	−0.080	0.013	−0.370	0.038	0.282	−0.147
*Methanoculleus*	17.34 ± 5.32b	106.57 ± 62.41a	3.98 ± 0.9b	12.69 ± 5.65b	7.88 ± 1.39b	−0.276	−0.221	−0.391	−0.186	−0.129	−0.402
***Synergistetes***											
*Aminobacterium*	3.84 ± 0.27	3.63 ± 1.58	1 ± 0.06	2.42 ± 0.56	2.09 ± 1.3	−0.448	−0.387	−0.582*	0.135	0.334	−0.738**
***Thermotogae***											
S1	1.08 ± 0.09	1.1 ± 0.25	0.76 ± 0.21	0.56 ± 0.08	0.71 ± 0.15	−0.586*	−0.604*	−0.395	0.458	0.341	−0.871**
***WWE1***											
W22	3.9 ± 1.85a	0.58 ± 0.05b	2.13 ± 0.36ab	0.81 ± 0.2b	0.91 ± 0.43b	−0.486	−0.397	−0.372	0.616*	0.643**	−0.673**

The data were based on 16S rDNA datasets

All data are presented as means ± standard deviations; values with different letters in a row mean significant difference at p < 0.05; bold italic fonts stand for phylum, while non-bold fonts stand for genus and 1 OTU

*DCP* daily CH4 production

** Significant at *p* < 0.01; * Significant at *p* < 0.05

However, there were some differences in between peak period and stable period. For example, in peak period (Table 4), the OTU84 was more active at 45 and 50 °C, and their potentially relative activities positively correlated with DCP. The genus S1 was more active at 45 and 50 °C, with positive correlation with DCP. Although the relative abundances of genus *Aminobacterium* were low in 16S rDNA datasets, their potentially relative activities were high at 50 °C.

**Table 4:** The relative activities of specific populations in peak period and their correlations to the performances

	25 °C	35 °C	45 °C	50 °C	55 °C	Temperature	DCP	Acetic acid	Propionic acid	Butyric acid	NH_4_^+^-N
***Firmicutes***											
Unclassified *Clostridiales*	0.8 ± 0.2a	0.6 ± 0.1ab	0.5 ± 0b	0.6 ± 0ab	0.5 ± 0.1ab	−0.534*	−0.427	0.512	0.366	0.560*	−0.390
Unclassified *Clostridiaceae*	0.2 ± 0b	0.2 ± 0b	0.4 ± 0.2ab	0.3 ± 0b	1.1 ± 0.5a	0.494	0.092	−0.204	−0.449	−0.226	0.699**
Unclassified *Lachnospiraceae*	0.2 ± 0b	0.8 ± 0.2a	0.4 ± 0b	0.4 ± 0b	0.5 ± 0ab	0.152	0.068	−0.530*	−0.318	−0.554*	0.225
*Lactobacillus*	2.3 ± 0.2b	2.5 ± 1.8b	3.1 ± 1.5b	1.1 ± 0.1b	9.9 ± 1.4a	0.469	−0.169	−0.218	−0.472	−0.216	0.521*
*Clostridium*	0.3 ± 0.1c	0.4 ± 0.1bc	0.5 ± 0bc	0.6 ± 0ab	0.8 ± 0.1a	0.767**	0.311	−0.521*	−0.828**	−0.563*	0.931**
*Sedimentibacter*	0.4 ± 0b	0.1 ± 0c	0.1 ± 0c	0.1 ± 0c	0.9 ± 0.2a	0.313	−0.334	0.111	−0.319	0.07	0.274
*Syntrophomonas*	0.6 ± 0.2a	0.1 ± 0b	0.2 ± 0.1b	0.2 ± 0.1b	0.2 ± 0.1b	−0.549*	−0.45	0.824**	0.633*	0.758**	−0.507
*Tepidimicrobium*	0.7 ± 0.3	1.5 ± 0.9	0.9 ± 0.1	0.3 ± 0.1	1.1 ± 0.3	−0.082	−0.236	0.063	0.069	−0.037	−0.092
*Pelotomaculum*	0.6 ± 0.2b	0.5 ± 0.3b	0.8 ± 0.2b	9.1 ± 4a	0.6 ± 0.4b	0.304	0.582*	−0.244	−0.345	−0.222	0.347
***Bacteroidetes***											
*Ruminofilibacter*	0.6 ± 0	1.2 ± 0.4	1.2 ± 0.2	2.9 ± 0.9	3 ± 2.4	0.435	0.247	−0.254	−0.424	−0.31	0.861**
OTU84	0.7 ± 0.1	0.8 ± 0.4	1 ± 0.2	1.2 ± 0.2	0.9 ± 0.2	0.298	0.516*	−0.145	−0.139	−0.222	0.355
Unclassified *Bacteroidales*	8.8 ± 0.4a	5.1 ± 2.1b	2.1 ± 0.1bc	0.4 ± 0c	0.4 ± 0c	−0.904**	−0.728**	0.865**	0.895**	0.832**	−0.986**
*Prevotella*	0.2 ± 0c	0.9 ± 0.4abc	1.6 ± 0.3a	0.7 ± 0.1bc	1.1 ± 0.1ab	0.463	0.41	−0.590*	−0.329	−0.558*	0.454
*Bacteroides*	1.4 ± 0.2b	5.1 ± 1.8a	3.4 ± 0.5ab	0.6 ± 0.1b	0.7 ± 0.1b	−0.316	−0.162	−0.193	0.21	−0.156	−0.305
Unclassified *Porphyromonadaceae*	0.3 ± 0	0.2 ± 0	0.3 ± 0	0.2 ± 0.1	0.3 ± 0.1	−0.049	−0.085	0.185	0.161	0.194	−0.047
***Euryarchaeota***											
*Methanosarcina*	2.5 ± 0.4	2.8 ± 1	1.6 ± 1.1	6.4 ± 1.3	11.3 ± 7.1	0.441	0.163	−0.590*	−0.472	−0.216	0.686**
*Methanoculleus*	13.8 ± 0.7b	93.7 ± 39.8a	38 ± 13.6ab	35.1 ± 8.6ab	10.7 ± 1.8b	−0.164	0.059	−0.138	0.087	−0.197	−0.167
*Methanobrevibacter*	0.6 ± 0.1	0.4 ± 0.2	0.3 ± 0.1	0.3 ± 0.1	0.6 ± 0.2	−0.124	−0.445	0.263	0.064	0.283	−0.302
*Methanobacterium*	1.1 ± 0.6	0.6 ± 0.2	0.9 ± 0.3	1.3 ± 0.5	2.4 ± 1	0.379	0.054	−0.194	−0.389	−0.142	0.599*
***Thermotogae***											
S1	0.7 ± 0b	1.4 ± 0.5b	3.1 ± 0.9a	3.7 ± 0.5a	0.7 ± 0.1b	0.332	0.746**	−0.479	−0.261	−0.44	0.409
***WWE1***											
W22	7.6 ± 0.7a	1.9 ± 0.4b	1.3 ± 0.4b	0.8 ± 0.2b	0.9 ± 0.1b	−0.848**	−0.733**	0.960**	0.855**	0.957**	−0.895**
***Synergistetes***											
*Aminobacterium*	0.7 ± 0.2b	1.7 ± 0.3ab	1.4 ± 0.2ab	4.7 ± 2.3a	1.8 ± 0.6ab	0.38	0.511	−0.388	−0.42	−0.374	0.536*

The data were based on 16S rDNA datasets

All data are presented as means ± standard deviations; values with different letters in a row mean significant difference at p < 0.05; bold italic fonts stand for phylum, while non-bold fonts stand for genus and 1 OTU

*DCP* daily CH4 production

** Significant at *p* < 0.01; * Significant at *p* < 0.05

### Predicted functional profiles of microbial communities

The functional profiles of microbial communities in peak and stable periods were predicted by PICRUSt (Additional file 1: Fig. S7). Overall, the predicted methanogenic metabolism (mainly referred to methanogenesis in AD systems) increased with temperature from 25 to 50 °C, and positively correlated with DCP (Additional file 2: Table S4). Predicted methanogenic activities based on 16S rRNA datasets were higher (*p* < 0.005) than those based on 16S rDNA datasets, which indicated more sensitivity of RNA molecule compared to DNA for predicting system function. NSTI value was 0.13 ± 0.01, indicating a good prediction for methanogenic metabolism [24].

### Microbial community influence fermentation performance

Gradual changes in temperature may have little effect on microbial community until a threshold is reached at which a large shift occurs [25]. In this study, each temperature resulted in a defined microbial community pattern, implicating that temperature changes by 5 °C could dramatically shift a microbial community in AD systems. The shifts of specific taxa under temperature gradient reflect their adaptations to different niches in AD process. Meanwhile, the shifts of microbial community also implicate that the relative contributions of different populations to the AD process change with temperature, which finally results in different efficiency in system functions.

Overall, Firmicutes, including main genera such as *Lactobacillus*, *Clostridium*, *Sedimentibacter* and *Tepidimicrobium*, were abundant at elevated temperature especially at 50 °C, which could accelerate hydrolytic activity for lipids, proteins and polymeric carbohydrates [4, 26, 27]. The phylum Bacteroidetes including cellulose-degrading communities were abundant at 25–45 °C, which agreed well with previous report that Bacteroidetes are major bacterial components in mesophilic biogas digesters [9]. However, some taxa (genus *Ruminofilibacter* and OTU84 *Ruminofilibacter xylanolyticum*) with the capability to degrade xylan [28, 29] showed different temperature-related patterns. They were significantly more abundant at 35–50 °C than those at 25 and 55 °C (*p* < 0.05). The phylum Euryarchaeota was more abundant at elevated temperature. Overall, both relative abundances and activities of *Methanosarcina* were high at elevated temperatures, implicating its increased contribution to methanogenesis at high temperature. Consequently, better fermentation performance especially for methane production at elevated temperatures was likely induced by higher methanogenesis and hydrolysis supported by discrepancy in abundances and activities of taxa discussed above and by PICRUSt prediction. However, they were not able to clarify the differences of performance between 50 and 55 °C. Given that most methanogens showed no significant difference in whole activities and abundances at between 50 and 55 °C, it was reasonably speculated that the discrepancy of performance was mainly caused by the hydrolysis steps. This was supported by the significant differences of the abundances and whole activities in dominant phylum Firmicutes and Bacteroidetes between 50 and 55 °C.

### The diversity of microbial community

**Fig. 4:**
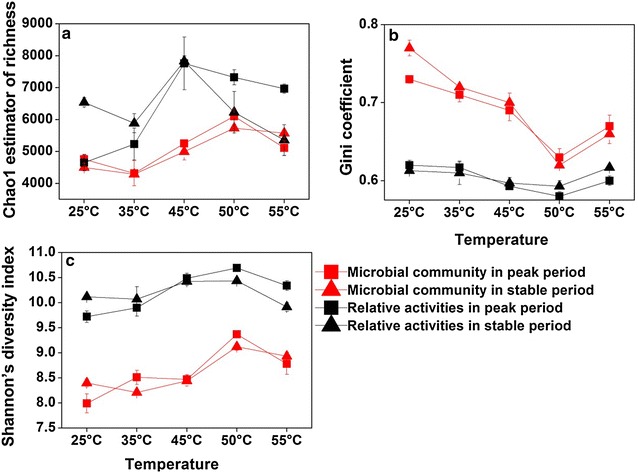
The alpha-diversity indices of microbial communities based on 16S rDNA datasets and of potentially relative activities of microbial populations at different temperatures. **a** Chao1 estimator of richness. **b** Evenness expressed by Gini coefficient. **c** Shannon’s diversity index. Overall, these indices positively correlated with temperature and DCP (*p* < 0.01). All the data are presented as means ± standard deviations (n = 3)

To further explore the microbial community structure, the alpha-diversity indices including Chao1 richness, Shannon’s diversity index and evenness expressed by Gini coefficient (with the low value meaning high evenness) were assessed in peak and stable period samples based on 16S rDNA datasets. Overall, these alpha-diversity indices increased with temperature from 25 to 50 °C, and decreased at 55 °C (Fig. 4). These alpha-diversity indices showed significant and positive correlations with DCP (n = 30, *p* < 0.01) (Additional file 1: Fig. S8).

The diversities of potentially relative activities of microbial populations obviously varied along the temperature gradient. The samples in each specific temperature usually formed a cluster (Additional file 1: Fig. S9) regardless of digestion period, implicating that temperature was key to drive the shifts of potentially relative activities of microbial populations. PERMANOVA tests further showed that the significant differences (*p* < 0.01) in potentially relative activities were detected at different temperatures, especially between those at 45 and 55 °C and those at 25 and 35 °C, which was similar with the variation in whole activities of microbial community. The alpha-diversity indices (Shannon’s diversity index, Chao1 richness and evenness) of potentially relative activities of microbial populations were assessed under temperature gradient combining both peak and stable period samples (Fig. 4). Results indicated that all indices showed linear correlations with DCP (*p* < 0.01) (Additional file 1: Fig. S10), which actually showed the similar correlation patterns as that between alpha-diversity of microbial community and DCP. The alpha-diversity of potentially relative activities of microbial populations increased with temperature from 25 to 50 °C, indicating more diverse and active metabolic pathways existing at elevated temperatures from 45 to 50 °C.

### The diversity of microbial community influence the fermentation performance

Increased hydrolysis rates with temperature are likely supported by higher microbial diversity and activity within a temperature range, so that an efficient performance could be maintained. However, when temperature is higher than a certain threshold, increased hydrolysis rates may cause high level NH_4_^+^-N, molecular hydrogen accumulation and other toxic compounds, which finally reduce microbial diversity and system stability. The temperature threshold for optimal performance of an AD system might be determined by the compounded effects of temperature-dependent shifts in microbial community structure, diversity and activity, reaction rate and inhibition factors. In AD systems with multiple syntrophic metabolism network [30], increased diversity with temperature might provide more functional redundancy with access to the total functional diversity and environmental specificity available in the community, thus enhancing the performance of biogas digesters [31, 32]. The high diversity provides community with more diverse metabolic pathways [32, 33]. In a community with high diversity, it maintains a dynamic balance on the production and consumption of metabolites along the trophic chain [34]. Thermodynamic difficulties might be conquered by multiple syntrophic metabolisms. Stability of an ecosystem is also crucial to maintain its function [35, 36]. It has indicated that parallel processing of substrate correlates with greater functional stability when methanogenic bioreactor communities are perturbed by glucose [32]. This study further showed that both the alpha-diversities of microbial communities and potentially relative activities were consistently and linearly correlated with DCP. The high alpha-diversity of microbial communities presents more potential redundancy of fermentative populations, and improves the stability of the AD system, such as a high resistance to disturbance caused by the loss of some functional populations. High alpha-diversity of potentially relative activities reflects more potential redundancy of metabolic activities and pathways, and increase in this diversity will enhance the metabolic efficiency. Both diversity indices reflect the functional redundancy. High functional redundancy is crucial for the stable and efficient performance in an AD system. Both diversity indices were higher at 50 °C than that at 55 °C, further explaining the discrepancy of performance between 50 and 55 °C. Consequently, high alpha-diversities regulated by temperature improved the methane production in the AD system.

### Contribution of temperature effects to AD performance

**Fig. 5:**
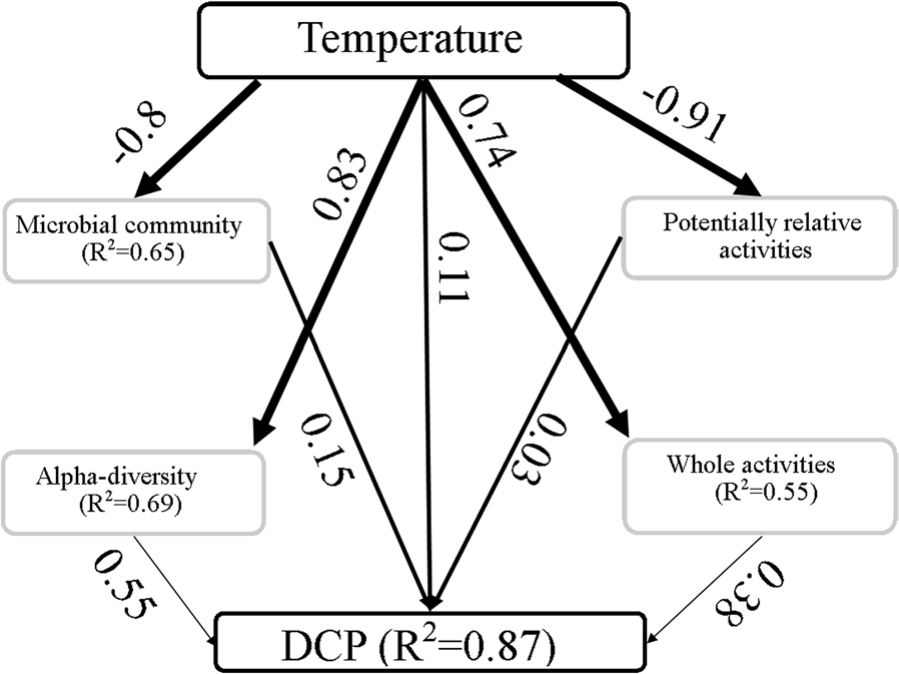
Partial Least Squares Path Modeling analysis of the effects of temperature on DCP. The values on the* line* mean the direct effects to the variables pointed by *arrow*. The GoF value is 0.68 in this model, roughly indicating that prediction power of the model is 68 %

Pearson’s correlation test showed that temperature correlated positively with DCP (*p* < 0.01) (Additional file 2: Table S4). However, direct and indirect contributions of temperature to DCP should be further explored and summarized. Partial Least Squares Path Modeling (PLS-PM) [37, 38] is a comprehensive model for analyzing multiple relationships between blocks of variables. PLS-PM was conducted to further detect how temperature affected the DCP using alpha-diversity (Chao1 index, Shannon index and evenness), whole activities, abundances and potentially relative activities of microbial communities, with data from both peak and stable periods. In the first level, the effect values from temperature on the alpha-diversity, whole activities, abundances and potentially relative activities of microbial communities, and DCP were 0.83, 0.74, −0.8, −0.91 and 0.11, respectively (Fig. 5), which reflected direct effects of temperature on above variables. In the second level, the alpha-diversity, whole activities, abundances and potentially relative activities of microbial communities directly affected DCP with effect values of 0.55, 0.38, 0.15 and 0.03, respectively. The total effects (including direct and indirect effect) on DCP from temperature was 0.70 with more indirect effects (0.59) than direct effects (0.11) (Additional file 2: Table S8). Among the indirect effects, 0.45, 0.29, −0.12 and −0.03 were from the alpha-diversity, whole activities, abundances and potentially relative activities of microbial communities, respectively. This further indicated that temperature affected the DCP mainly through regulating the alpha-diversity and whole activities of microbial communities. Partial mantel tests were conducted to further verify the above results (Additional file 2: Table S9), showing that whole activities and alpha-diversity of microbial community contributed much to the variation of DCP.

## Conclusions

This study revealed how microbial community (composition, structure, activity) responded to temperatures, and the microbial mechanisms of temperature regulation on AD performance. For the anaerobic digestion of swine manure, 50 °C was likely a threshold below which methane production rates increased linearly with temperature. The variations of potentially relative activities of specific populations and those of whole activities of microbial community under temperature gradient were firstly revealed, which provided a new insight to assess the roles of specific microorganisms, and crucially uncovered the importance of whole activities of microbial community for fermentation performance. Besides, increased alpha-diversity of both microbial communities and potentially relative activities supplied more functional pathways and activities for metabolism network in AD systems. Consequently, the whole activities and alpha-diversity of microbial community could be robust indicators for fermentation performance especially for methane production in AD systems.

## Methods

### Setup of biogas digestion system

The anaerobic digestion experiment was set up in a 2 L anaerobic flask containing 1.5 L digestion slurry with final total solid (TS) content of 8 % (Additional file 2: Table S1). The anaerobic flasks were designed to own two holes on the upper and lower flask-wall. Feedstock and discharge were through the two holes respectively by peristaltic pump (Cat. NO. BT50s, Leadfluid, China). Four hundred and fifty millilitre seed slurry (TS 8 %) was inoculated at the start of digestion. Seed slurry was prepared by anaerobic digestion of swine manure (obtained from a pig farm in Chengdu, Sichuan Province, China) under respective experimental temperature, during which semi-continuous mode with HRT of 30 days was set. It ensured that each treatment group ran at least one time the HRT, and the digestion performance maintained a dynamic equilibrium with more than 60 % CH_4_ in produced biogas. The anaerobic digestion experiments were set up at 25, 35, 45, 50 and 55 °C. Triplicate reactors were set up for each temperature. After daily CH_4_ production (DCP) reached its first peak in a reactor, semi-continuous mode was made that 150 mL digestion slurry was replaced with same volume of fresh swine manure slurry (TS 8 %) every 3 days [Organic loading rate (ORL), 2 g VS L^−1^ day^−1^; Hydraulic retention time (HRT) 30 days] so that the fermentation process could maintain a dynamic equilibrium (stable period). Gyrated the flask with hand twice a day to mix thoroughly the fermentation content in a flask. This feeding pattern was determined based on pre-experimental results. Details about parameters at the start of fermentation were shown in Additional file 2: Table S1.

### Sampling and chemical analysis

The slurry samples were collected at day 1 (initial period, 24 h after inoculation, each sample 40 mL slurry, labeled as 25I, 35I, 45I, 50I, 55I respectively), peak I (the time varied based on temperature, labeled as 25P, 35P, 45P, 50P, 55P respectively), and stable period (48 h after second slurry change, labeled as 25S, 35S, 45S, 50S, 55S respectively) (details in Additional file 2: Table S2). First peak reflected the full potential of biogas production, while stable period represented the dynamic equilibrium during AD process. Centrifugation at 13,400×*g* for 5 min was used to pellet the slurry immediately for DNA and RNA extraction. The filtered supernatant with a 0.22 μm filter (Cat. NO. SLGP033RS; Millipore, USA) was used for chemical analysis. The VFAs in the supernatant were detected by High Performance Liquid Chromatography (HPLC, Agilent 1260), equipped with a column Hi-Plex H (300  ×  6.5 mm) and a differential refraction detector. The H_2_SO_4_ (0.005 M) with a flow rate of 0.6 mL min^−1^ was mobile phase; NH_4_^+^-N was quantified using Nessler’s reagent colorimetric method [39]. The biogas production was measured by water replacement method, and all the water replacement equipment were set under same room temperature (about 22 °C) and air pressure (about 95.86 kpa) outside the temperature-controlled incubators, which avoided the bias of measured volume induced by different temperatures and pressures. CH_4_ and H_2_ were measured by gas chromatography system (Agilent 6890 system: Argon carrier gas at 30 mL min^−1^) which is equipped with a 2 m stainless steel column packed with 143 Porapak Q (50/80 mesh). The finally displayed volume of these gas had been normalized at standard temperature (273 K) and pressure (101325 Pa) based on the ideal gas law [40, 41]. TS, VS and COD were measured as described previously [42].

### DNA and RNA extraction and 16S rRNA gene amplicon sequencing

The Ezup Column Soil DNA Purification Kit (Cat. No. B518263; Sangon Biotech, China) was used for total DNA extraction. The RNAprep pure Cell/Bacteria Kit (Cat. No. DP430; TIANGEN, China) was used for total RNA extraction. The reverse transcription kit (Cat. No. PR6901; Thermo, USA) was used for complimentary DNA (cDNA) synthesis. The 16S rRNA gene was amplified from DNA and cDNA with universal primers 515F (5′-GTGCCAGCMGCCGCGGTAA-3′) and 806R (5′-GGACTACHVGGGTWTCTAAT-3′) (for both bacteria and archaea). Two parallel 25 µL PCR reactions were conducted, and the PCR products were pooled for purification by the method of electrophoresis. The details in PCR procedure and sample preparation were described before [43]. In total, 90 samples were prepared for sequencing on the Illumina Miseq platform.

### Miseq sequence data analysis

Amplicon sequence analysis was conducted by QIIME Pipeline Version 1.7.0 (http://www.qiime.org/tutorials/tutorial.html) [44]. All sequence reads were sorted with their unique barcodes. UCHIME algorithm was used for the removal of chimera sequences [45]. A 97 % identity of cutoff was used to cluster sequences into OTUs. Daisychopper.pl (http://www.festinalente.me/bioinf/downloads/daisychopper.pl) was used to resample the sequences to the same sequence depth (7710 reads per sample) for downstream analysis. The phylogenetic affiliation of each sequence was analyzed by the Ribosomal Database Project classifier [46].

### Statistical analysis

The general changes of microbial community structure with temperatures were assessed by principal coordinates analysis (PCoA) and PERMANOVA which were performed in R (http://www.r-project.org/) based on Bray-Curtis distance. The SPSS 21 software (IBM USA) was performed to evaluate the normality and homoscedasticity of the data. The differences in relative abundances of taxonomic units between samples at different temperatures were tested by One-way-analysis of variance (ANOVA) performed in SPSS 21 software. Pearson’s correlation analysis and Partial Mantel Tests were performed in R to assess the correlation between variables. Regression analysis was conducted using OriginPro 8.5 software (OriginLab USA). A computational approach, phylogenetic investigation of communities by reconstruction of unobserved states (PICRUSt) [24], was used to predict functional profiles of microbial communities in the AD system using both 16S rDNA and 16S rRNA datasets. Partial Least Squares Path Modeling was performed in R to assess direct and indirect effects of temperature on AD performance. The GoF index measures the overall quality at both the measurement and the structural models [37].

## Additional files

10.1186/s12934-016-0491-x Additional figures.
10.1186/s12934-016-0491-x Additional tables.

## Abbreviations

AD: anaerobic digestion
VFAs: volatile fatty acids
DCP: daily CH_4_ production (L L^−1^ day^−1^)
TBP: total biogas production in 27 days (L kg^−1^ VS)
ACP: average CH_4_ production (L kg^−1^ VS day^−1^) from initial period to peak period
PCoA: principal coordinates analysis
HPLC: high performance liquid chromatography
OTU: operational taxonomic unit
ANOVA: one-way-analysis of variance
PERMANOVA: permutational multivariate analysis of variance
HRT: hydraulic retention time
OLR: organic loading rate
VS: volatile solid
PLS-PM: partial least squares path modeling
HRT: hydraulic retention time
OLR: organic loading rate
VS: volatile solid

## References

[1] Weiland P (2010). Biogas production: current state and perspectives. Appl Microbiol Biotechnol.

[2] Rui J, Li J, Zhang S, Yan X, Wang Y, Li X (2015). The core populations and co-occurrence patterns of prokaryotic communities in household biogas digesters. Biotechnol Biofuels.

[3] Labatut RA, Angenent LT, Scott NR (2014). Conventional mesophilic vs. thermophilic anaerobic digestion: a trade-off between performance and stability?. Water Res.

[4] De Vrieze J, Saunders AM, He Y, Fang J, Nielsen PH, Verstraete W, Boon N (2015). Ammonia and temperature determine potential clustering in the anaerobic digestion microbiome. Water Res.

[5] Zhang W, Werner JJ, Agler MT, Angenent LT (2014). Substrate type drives variation in reactor microbiomes of anaerobic digesters. Bioresour Technol.

[6] Wilson CA, Murthy SM, Fang Y, Novak JT (2008). The effect of temperature on the performance and stability of thermophilic anaerobic digestion. Water Sci Technol.

[7] Boske J, Wirth B, Garlipp F, Mumme J, Van den Weghe H (2015). Upflow anaerobic solid-state (UASS) digestion of horse manure: thermophilic vs. mesophilic performance. Bioresour Technol.

[8] Gou C, Yang Z, Huang J, Wang H, Xu H, Wang L (2014). Effects of temperature and organic loading rate on the performance and microbial community of anaerobic co-digestion of waste activated sludge and food waste. Chemosphere.

[9] Pap B, Gyoerkei A, Boboescu IZ, Nagy IK, Biro T, Kondorosi E, Maroti G (2015). Temperature-dependent transformation of biogas-producing microbial communities points to the increased importance of hydrogenotrophic methanogenesis under thermophilic operation. Bioresour Technol.

[10] Angenent LT, Karim K, Al-Dahhan MH, Domiguez-Espinosa R (2004). Production of bioenergy and biochemicals from industrial and agricultural wastewater. Trends Biotechnol.

[11] Demirel B, Scherer P, Yenigun O, Onay TT (2010). Production of methane and hydrogen from biomass through conventional and high-rate anaerobic digestion processes. Crit Rev Environ Sci Technol.

[12] Noll M, Klose M, Conrad R (2010). Effect of temperature change on the composition of the bacterial and archaeal community potentially involved in the turnover of acetate and propionate in methanogenic rice field soil. FEMS Microbiol Ecol.

[13] Gentile G, Giuliano L, D’Auria G, Smedile F, Azzaro M, De Domenico M, Yakimov MM (2006). Study of bacterial communities in Antarctic coastal waters by a combination of 16S rRNA and 16S rDNA sequencing. Environ Microbiol.

[14] Kamke J, Taylor MW, Schmitt S (2010). Activity profiles for marine sponge-associated bacteria obtained by 16S rRNA vs 16S rRNA gene comparisons. ISME J.

[15] Campbell BJ, Kirchman DL (2013). Bacterial diversity, community structure and potential growth rates along an estuarine salinity gradient. ISME J.

[16] Acinas SG, Marcelino LA, Klepac-Ceraj V, Polz MF (2004). Divergence and redundancy of 16S rRNA sequences in genomes with multiple rrn operons. J Bacteriol.

[17] Hugoni M, Taib N, Debroas D, Domaizon I, Dufournel IJ, Bronner G, Salter I, Agogue H, Mary I, Galand PE (2013). Structure of the rare archaeal biosphere and seasonal dynamics of active ecotypes in surface coastal waters. Proc Natl Acad Sci USA.

[18] Brettar I, Christen R, Hofle MG (2012). Analysis of bacterial core communities in the central Baltic by comparative RNA–DNA-based fingerprinting provides links to structure-function relationships. ISME J.

[19] Campbell BJ, Yu LY, Heidelberg JF, Kirchman DL (2011). Activity of abundant and rare bacteria in a coastal ocean. Proc Natl Acad Sci USA.

[20] De Vrieze J, Hennebel T, Boon N, Verstraete W (2012). Methanosarcina: the rediscovered methanogen for heavy duty biomethanation. Bioresour Technol.

[21] Zhang Y, Zamudio Canas EM, Zhu ZW, Linville JL, Chen S, He Q (2011). Robustness of archaeal populations in anaerobic co-digestion of dairy and poultry wastes. Bioresour Technol.

[22] Rajagopal R, Masse DI, Singh G (2013). A critical review on inhibition of anaerobic digestion process by excess ammonia. Bioresour Technol.

[23] Hunt DE, Lin Y, Church MJ, Karl DM, Tringe SG, Izzo LK, Johnson ZI (2013). Relationship between abundance and specific activity of bacterioplankton in open ocean surface waters. Appl Environ Microbiol.

[24] Langille MG, Zaneveld J, Caporaso JG, McDonald D, Knights D, Reyes JA, Clemente JC, Burkepile DE, Vega Thurber RL, Knight R (2013). Predictive functional profiling of microbial communities using 16S rRNA marker gene sequences. Nat Biotechnol.

[25] Scheffer M, Carpenter SR (2003). Catastrophic regime shifts in ecosystems: linking theory to observation. Trends Ecol Evol.

[26] Sundberg C, Al-Soud WA, Larsson M, Alm E, Yekta SS, Svensson BH, Sorensen SJ, Karlsson A (2013). 454 pyrosequencing analyses of bacterial and archaeal richness in 21 full-scale biogas digesters. FEMS Microbiol Ecol.

[27] Nelson MC, Morrison M, Yu Z (2011). A meta-analysis of the microbial diversity observed in anaerobic digesters. Bioresour Technol.

[28] Krober M, Bekel T, Diaz NN, Goesmann A, Jaenicke S, Krause L, Miller D, Runte KJ, Viehover P, Puhler A (2009). Phylogenetic characterization of a biogas plant microbial community integrating clone library 16S-rDNA sequences and metagenome sequence data obtained by 454-pyrosequencing. J Biotechnol.

[29] Weiss S, Zankel A, Lebuhn M, Petrak S, Somitsch W, Guebitz GM (2011). Investigation of mircroorganisms colonising activated zeolites during anaerobic biogas production from grass silage. Bioresour Technol.

[30] Lykidis A, Chen C-L, Tringe SG, McHardy AC, Copeland A, Kyrpides NC, Hugenholtz P, Macarie H, Olmos A, Monroy O (2011). Multiple syntrophic interactions in a terephthalate-degrading methanogenic consortium. ISME J.

[31] Hashsham SA, Fernandez AS, Dollhopf SL, Dazzo FB, Hickey RF, Tiedje JM, Criddle CS (2000). Parallel processing of substrate correlates with greater functional stability in methanogenic bioreactor communities perturbed by glucose. Appl Environ Microbiol.

[32] Werner JJ, Knights D, Garcia ML, Scalfone NB, Smith S, Yarasheski K, Cummings TA, Beers AR, Knight R, Angenent LT (2011). Bacterial community structures are unique and resilient in full-scale bioenergy systems. Proc Natl Acad Sci USA.

[33] Wittebolle L, Marzorati M, Clement L, Balloi A, Daffonchio D, Heylen K, De Vos P, Verstraete W, Boon N (2009). Initial community evenness favours functionality under selective stress. Nature.

[34] Fernandez A, Huang SY, Seston S, Xing J, Hickey R, Criddle C, Tiedje J (1999). How stable is stable? Function versus community composition. Appl Environ Microbiol.

[35] Ives AR, Carpenter SR (2007). Stability and diversity of ecosystems. Science.

[36] Pimm SL (1984). The complexity and stability of ecosystems. Nature.

[37] Tenenhaus M, Vinzi VE, Chatelin YM, Lauro C (2005). PLS path modeling. Comput Stat Data Anal.

[38] Henseler J, Chin WW (2010). A comparison of approaches for the analysis of interaction effects between latent variables using partial least squares path modeling. Struct Equ Model.

[39] Hart SC, Stark JM, Davidson EA, Firestone MK. Nitrogen mineralization, immobilization, and nitrification. In: methods of soil analysis: part 2—microbiological and biochemical properties. 1994(methodsofsoilan2). p. 985–1018.

[40] Uhlenbeck GE, Gropper L (1932). The equation of state of a non-ideal Einstein-Bose or Fermi-Dirac gas. Phys Rev.

[41] Bludman SA, Vanriper KA (1977). Equation of state of an ideal ferni gas. Astrophys J.

[42] APHA, editor. Standard methods for the examination of water and wastewater. Washington, DC: APHA; 1998.

[43] Li XZ, Rui JP, Mao YJ, Yannarell A, Mackie R (2014). Dynamics of the bacterial community structure in the rhizosphere of a maize cultivar. Soil Biol Biochem.

[44] Caporaso JG, Kuczynski J, Stombaugh J, Bittinger K, Bushman FD, Costello EK, Fierer N, Pena AG, Goodrich JK, Gordon JI (2010). QIIME allows analysis of high-throughput community sequencing data. Nat Methods.

[45] Edgar RC, Haas BJ, Clemente JC, Quince C, Knight R (2011). UCHIME improves sensitivity and speed of chimera detection. Bioinformatics.

[46] Wang Q, Garrity GM, Tiedje JM, Cole JR (2007). Naive Bayesian classifier for rapid assignment of rRNA sequences into the new bacterial taxonomy. Appl Environ Microbiol.

