# Supplementary material for: Microorganism-regulated mechanisms of temperature effects on the performance of anaerobic digestion
Source: Microb Cell Fact. 2016 Jun 3;15:96. doi: 10.1186/s12934-016-0491-x (PMC4893225; doi:10.1186/s12934-016-0491-x)
Supplement: Supplementary file 1 — 10.1186/s12934-016-0491-x Additional figures. [file 12934_2016_491_MOESM1_ESM.docx]

**Supplementary Figures**

**
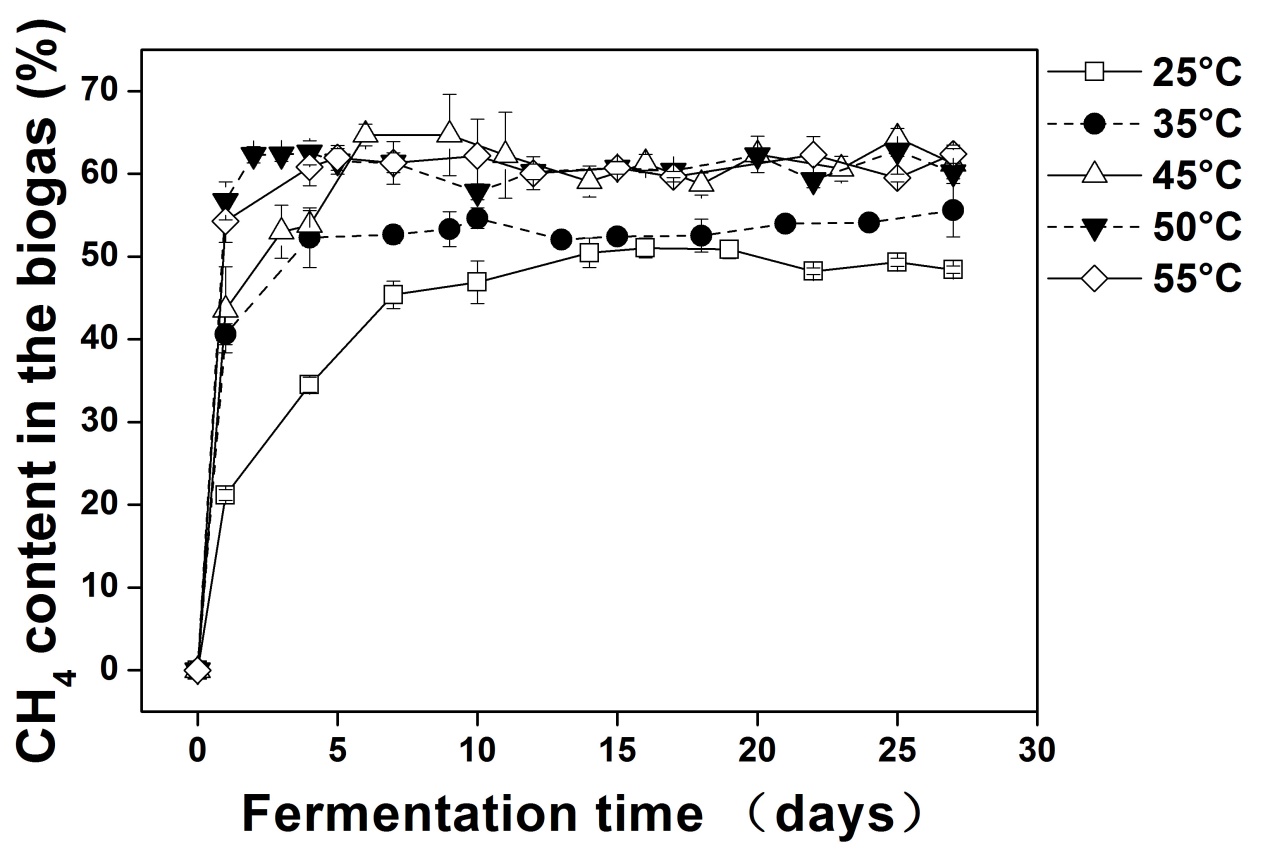
**

**Fig.S1** CH_4_ content in the biogas during fermentation process under all temperatures (25 to 55 °C). All the data are presented as means ± standard deviations (n=3).

**
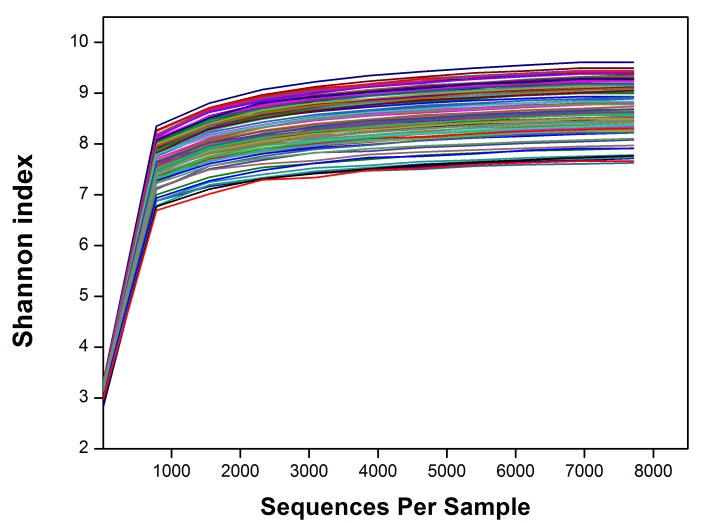
**

**Fig.S2** Rarefaction curves for 16S rDNA and 16S rRNA datasets under all temperatures (25 to 55 °C).


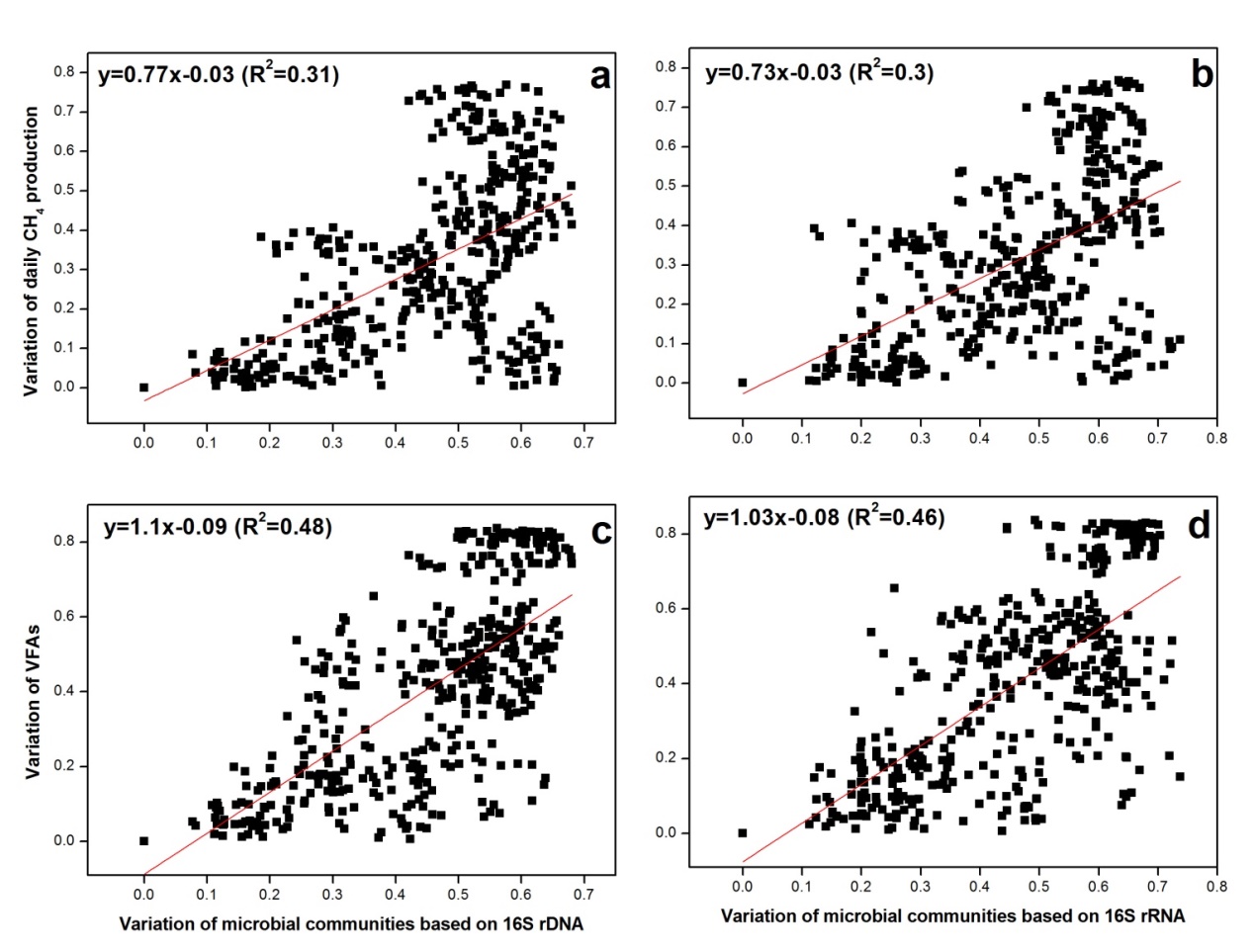


**Fig.S3** The relationships between the variation of microbial community and the variation of performance of AD system. “a” and “c” are based on 16S rDNA; “b” and “d” are based on 16S rRNA. The data used in analysis are from peak and stable periods. The red line is linearly fitting.

**
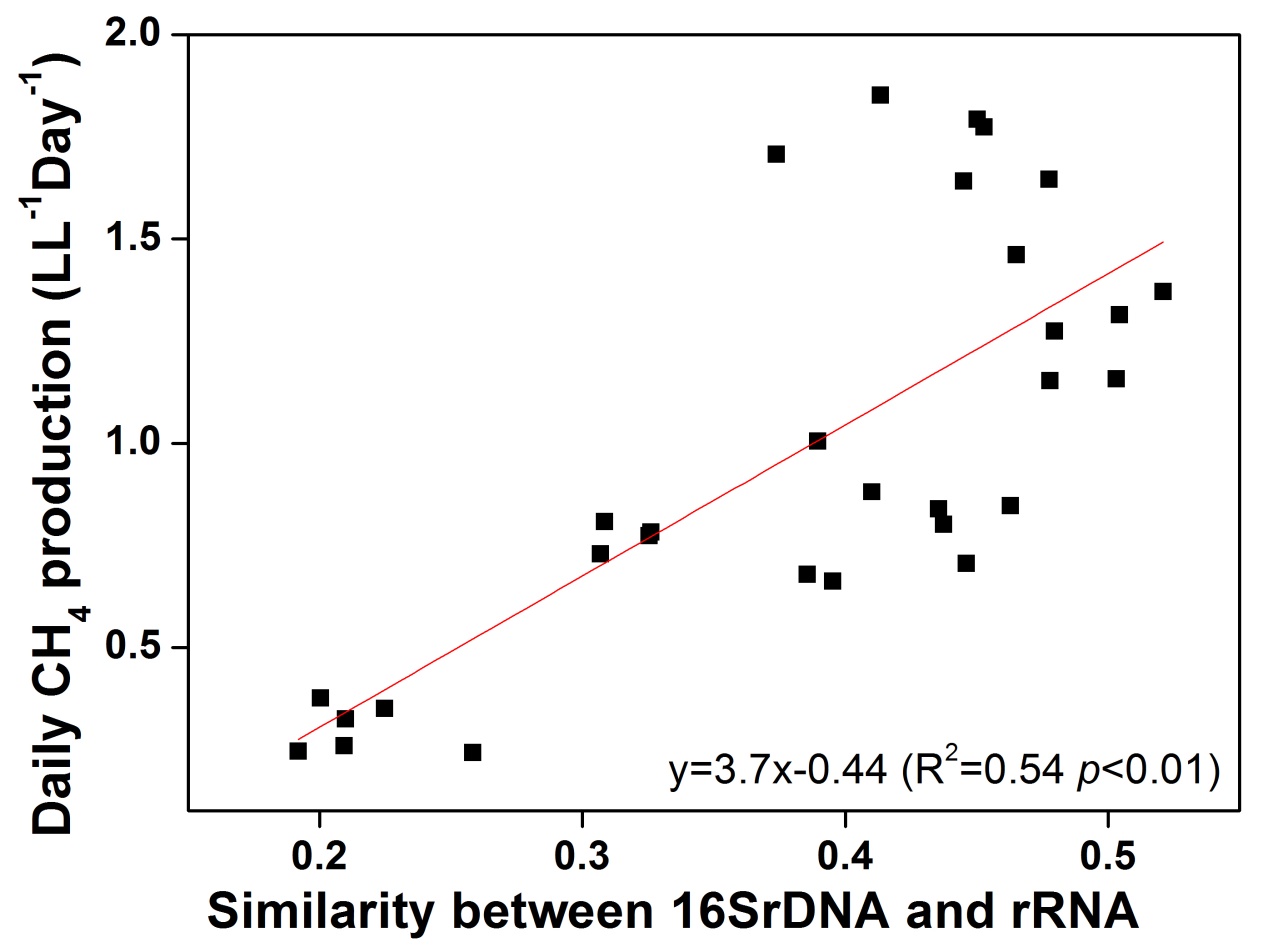
**

**Fig.S4** The correlation between daily CH_4_ production and the similarities between based on 16S rDNA and rRNA in both peak and stable periods. The red line is linearly fitting.


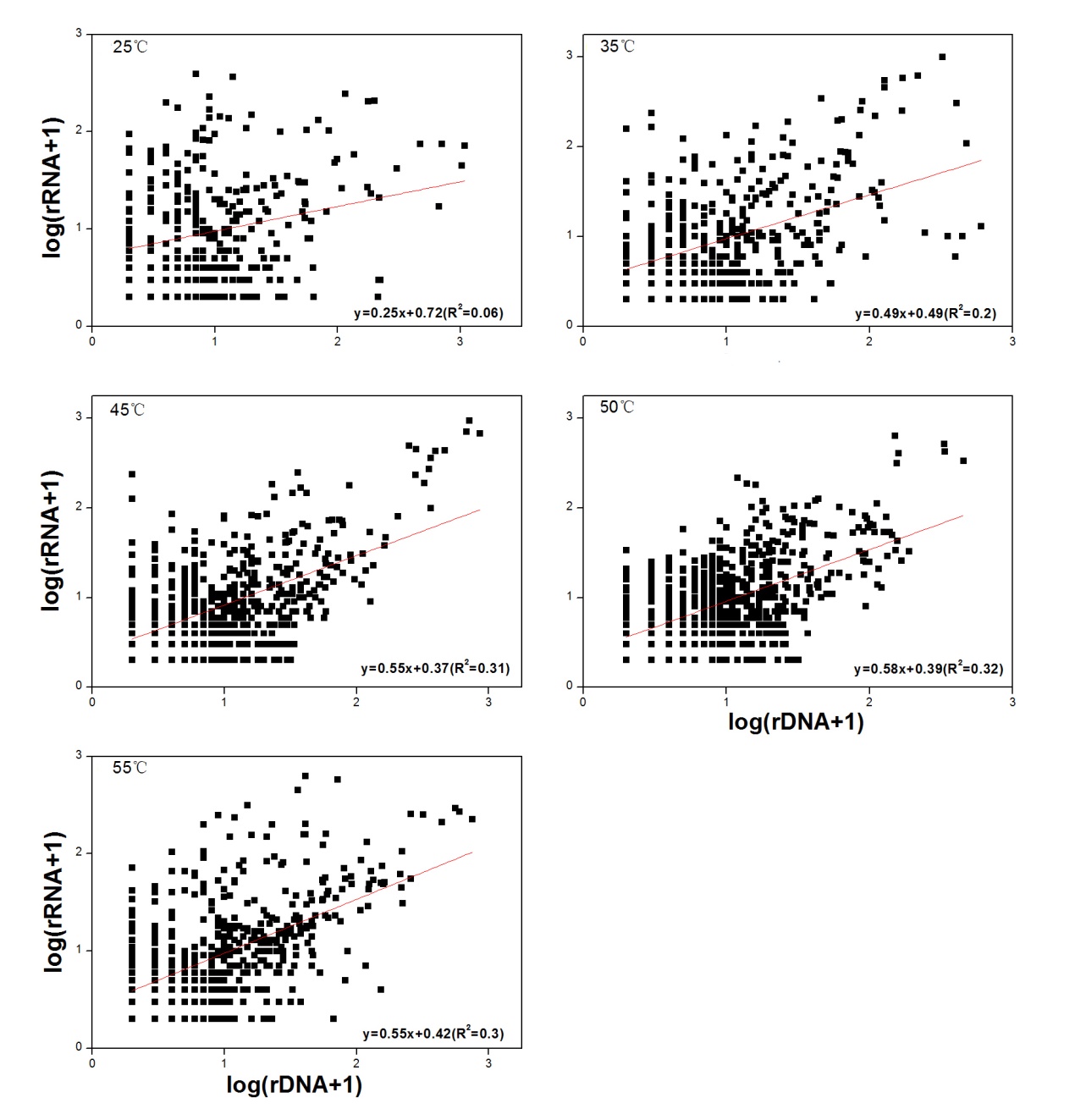


**Fig.S5** Relationship between activity [log (rRNA +1)] and abundance [log (rDNA +1)] of each OTU in stable period at different temperatures. To eliminate bias, correlations are made based on log (rRNA +1) and log (rDNA +1). Only the OTUs occurring in both 16S rRNA and 16S rDNA datasets are present. The red line is linear fitting.


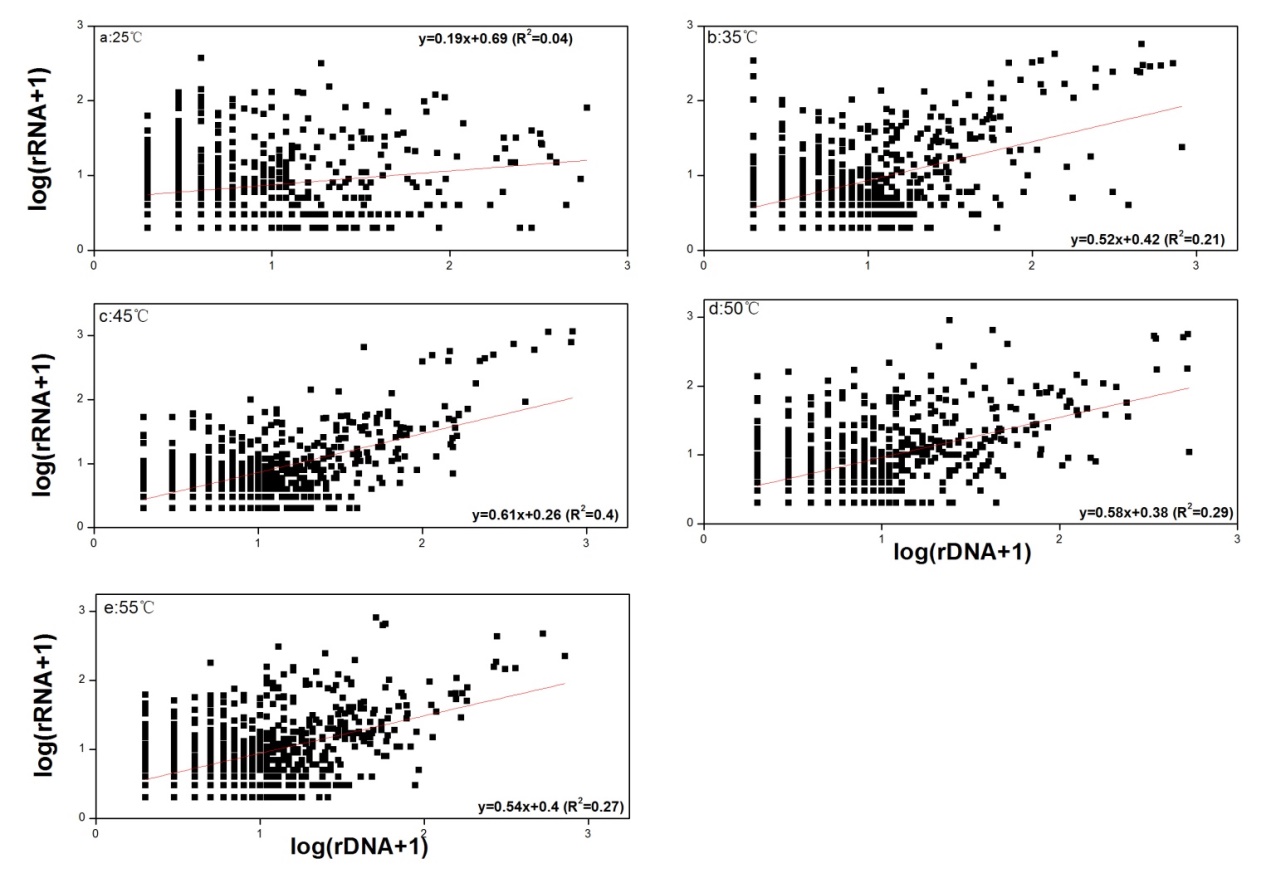


**Fig.S6** Relationship between activity [log (rRNA +1)] and abundance [log (rDNA +1)] of each OTU in peak period along temperature gradient. To eliminate bias, correlations are made based on log (rRNA +1) and log (rDNA +1). Only the OTUs occurring in both 16S rRNA and 16S rDNA datasets are present. The red line is linear fitting.

**
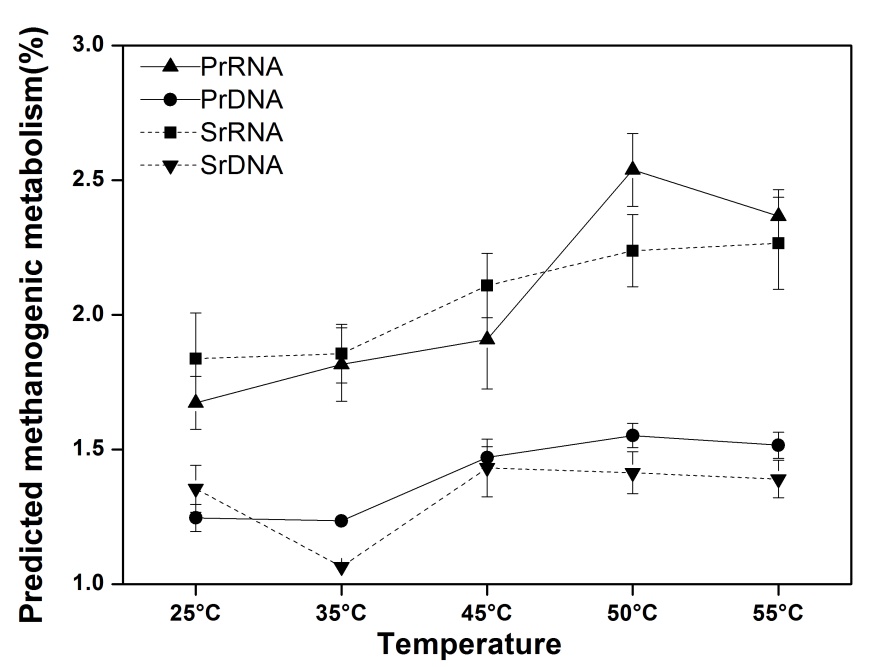
**

**Fig.S7** Prediction of methanogenic metabolism based on PICRUSt at different temperature. PrRNA: prediction based on 16S rRNA datasets in peak period; PrDNA: prediction based on 16S rDNA datasets in peak period; SrRNA: prediction based on 16S rRNA datasets in stable period; SrDNA: prediction based on 16S rDNA datasets in stable period.


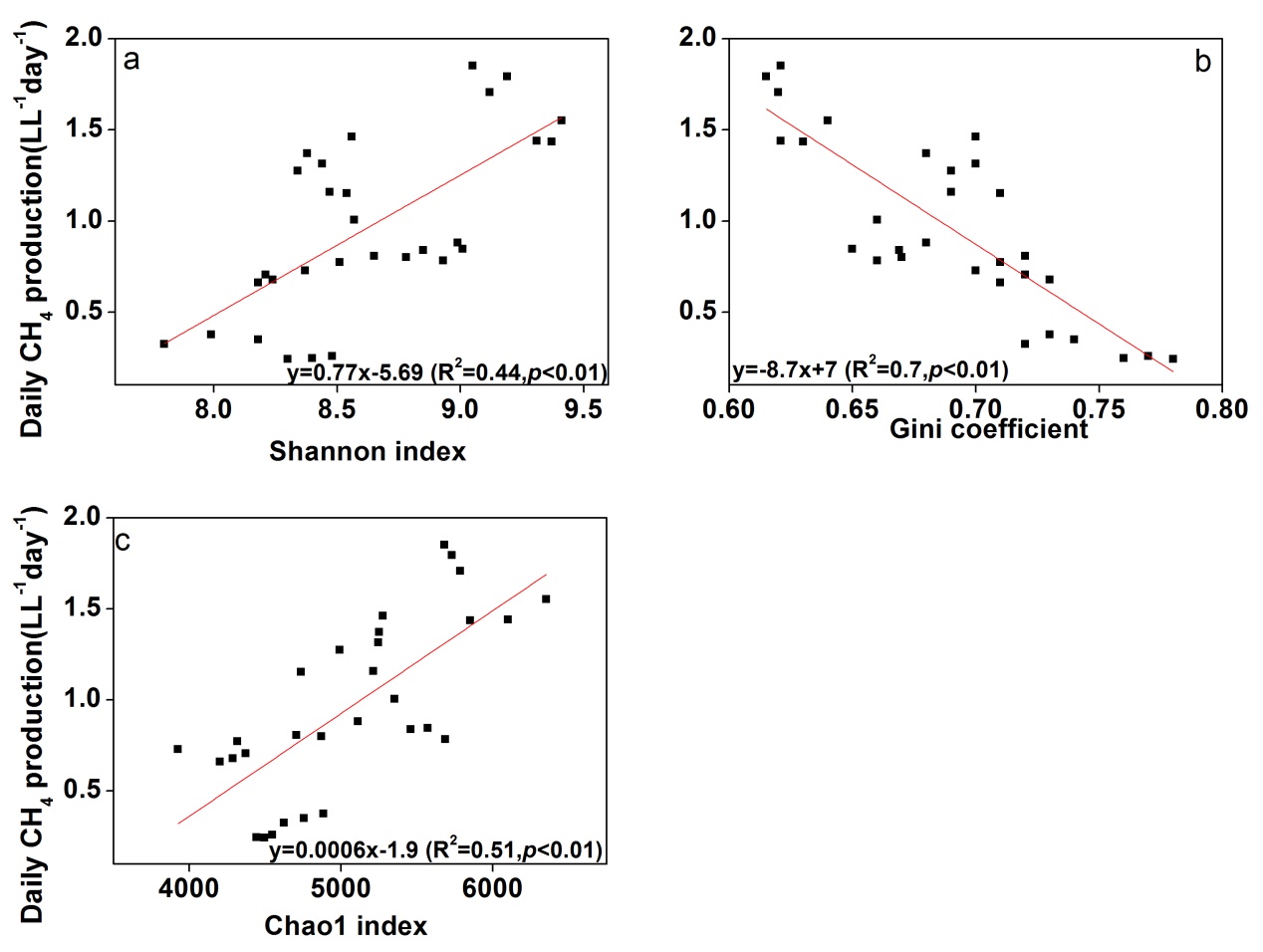


**Fig.S8** Relationships between alpha-diversity based on 16S rDNA datasets in peak and stable periods and daily CH_4_ production. The red line is linear fitting.


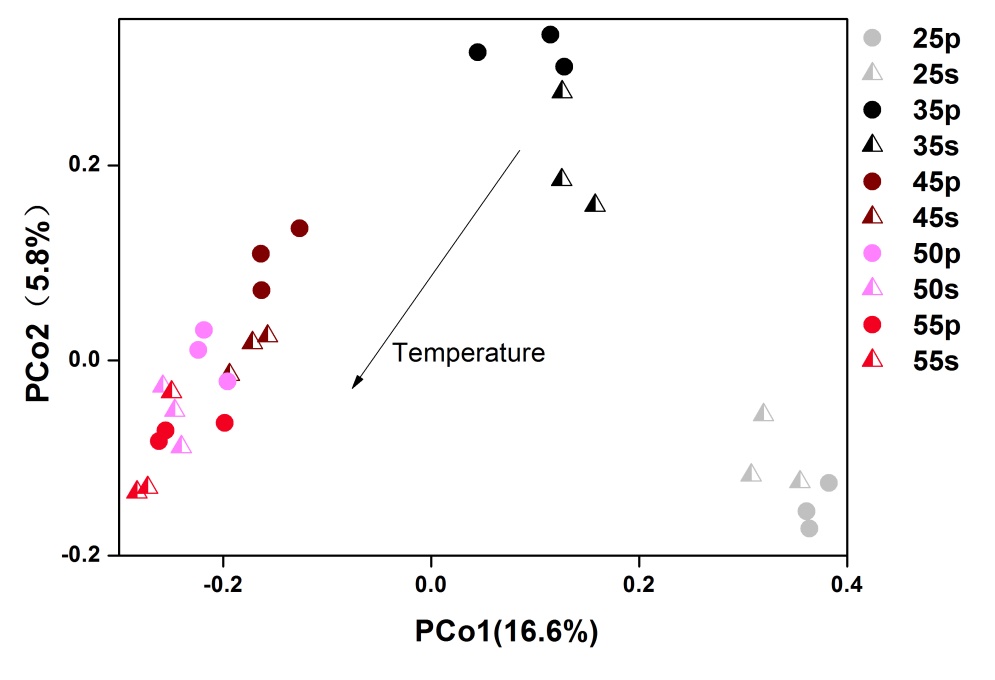


**Fig.S9** The principal coordinates analysis (PCoA) based on the potentially relative activities of microbial populations in peak and stable periods at different temperatures.


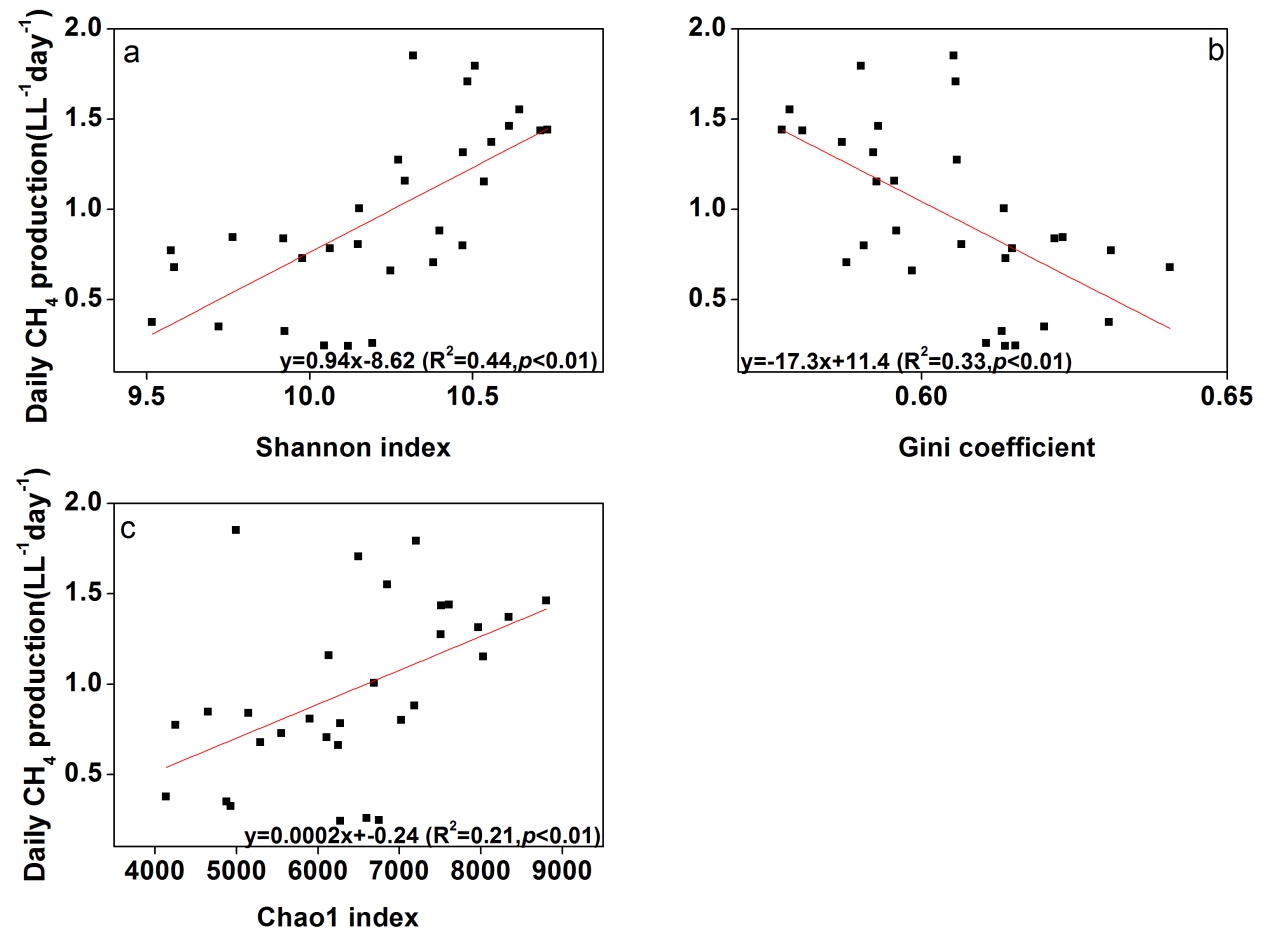


**Fig.S10** Relationships between alpha-diversity of potentially relative activities of microbial populations and daily CH_4_ production in the peak and stable period along temperature gradient.
